# Neuronal autosis is Na^+^/K^+^-ATPase alpha 3-dependent and involved in hypoxic-ischemic neuronal death

**DOI:** 10.1038/s41419-024-06750-2

**Published:** 2024-05-25

**Authors:** Pauline Depierre, Vanessa Ginet, Anita C. Truttmann, Julien Puyal

**Affiliations:** 1 Department of Fundamental Neurosciences, University of Lausanne, Lausanne, Switzerland; 2 Clinic of Neonatology, Department of Women, Mother and Child, University Hospital Center of Vaud, Lausanne, Switzerland; 3 CURML, University Center of Legal Medicine, Lausanne University Hospital, Lausanne, Switzerland

**Keywords:** Cerebrovascular disorders, Brain injuries

## Abstract

Macroautophagy (hereafter called autophagy) is an essential physiological process of degradation of organelles and long-lived proteins. The discovery of autosis, a Na^+^/K^+^-ATPase (ATP1)-dependent type of autophagic cell death with specific morphological and biochemical features, has strongly contributed to the acceptance of a pro-death role of autophagy. However, the occurrence and relevance of autosis in neurons has never been clearly investigated, whereas we previously provided evidence that autophagy mechanisms could be involved in neuronal death in different in vitro and in vivo rodent models of hypoxia-ischemia (HI) and that morphological features of autosis were observed in dying neurons following rat perinatal cerebral HI. In the present study, we demonstrated that neuronal autosis could occur in primary cortical neurons using two different stimulations enhancing autophagy flux and neuronal death: a neurotoxic concentration of Tat-BECN1 (an autophagy-inducing peptide) and a hypoxic/excitotoxic stimulus (mimicking neuronal death induced by cerebral HI). Both stimulations induce autophagic neuronal death (dependent on canonical autophagic genes and independent on apoptotic, necroptotic or ferroptotic pathways) with all morphological and biochemical (ATP1a-dependent) features of autosis. However, we demonstrated that autosis is not dependent on the ubiquitous subunit ATP1a1 in neurons, as in dividing cell types, but on the neuronal specific ATP1a3 subunit. We also provided evidence that, in different in vitro and in vivo models where autosis is induced, ATP1a3-BECN1 interaction is increased and prevented by cardiac glycosides treatment. Interestingly, an increase in ATP1a3-BECN1 interaction is also detected in dying neurons in the autoptic brains of human newborns with severe hypoxic-ischemic encephalopathy (HIE). Altogether, these results suggest that ATP1a3-BECN1-dependent autosis could play an important role in neuronal death in HI conditions, paving the way for the development of new neuroprotective strategies in hypoxic-ischemic conditions including in severe case of human HIE.

## Introduction

Macroautophagy (hereafter referred as autophagy), a highly conserved and physiological mechanism specialized in the delivery of intracellular material for lysosomal degradation [1–3], is characterized by the isolation of a membrane (phagophore) which elongates and incurves to engulf cytoplasmic content (such as long-lived proteins and organelles) in a multi-membrane vesicle termed autophagosome. Cargo degradation will then occur after the fusion of this intermediate compartment with a lysosome generating a third structure called autolysosome [4, 5]. An efficient flux of this multistep process is then essential for the maintenance of cell homeostasis and a failure in one of the different phases could result in various pathological conditions. Moreover, the level of autophagy flux can be adjusted to a variety of environmental or cellular stresses for cell survival. For a long time, the wide range of the fundamental roles played by autophagy in cell homeostasis, adaptation, and defense, have largely contributed to support the dogma that autophagy could only promote positive effects on cell health. However, after a period of intense debates [6–9] and important efforts to find a consensus on terminology and on experimental criteria [10, 11], the existence of detrimental roles of autophagy mechanisms in directly or indirectly (mediating through other cell death type) causing cell death has finally been recognized to occur in some specific conditions.

Nevertheless, some controversies remain concerning the existence and the relevance of a direct and essential role of autophagy in cell death that are fueled by the difficulties to establish a direct link between autophagy machinery and cell death processes, and to determine how (and which) autophagic mechanisms are subverted to damage the cell instead of protecting it. This is particularly important for neurons that are long-lived non-dividing cells with a high-energy demand and requiring strict homeostatic controls.

However, strong evidence for the existence of autophagic cell death in mammalian cells came from the recent discovery of autosis, a specific type of autophagic cell death [12]. Autosis was first observed and described in HeLa cells treated with the autophagy-inducing peptide Tat-BECLIN1 (Tat-BECN1) [13]. Tat-BECN1-induced autosis meets all the criteria for autophagic cell death including protection by pharmacological or genetical autophagy inhibition and complete independence of other types of regulated cell death [12]. However, in addition of an accumulation of autophagosomes and autolysosomes during a first phase (phase 1a), as during classical autophagic cell death, autosis presents highly specific morphological features in later phases (phases 1b and 2) resulting in a focal swelling of the perinuclear space (PNS) and the dilation and fragmentation of the endoplasmic reticulum. Moreover, autosis has been also biochemically characterized by its dependency on Na^+^K^+^-ATPase (ATP1) [12, 14–16]. In fact, cardiac glycosides, which are ligands of the alpha subunit of ATP1 (ATP1a) [16], have been shown to be potent autosis inhibitors and deletion or downregulation of ATP1a can prevent autosis in different cell types [12, 14, 17–19]. In addition to Tat-BECN1 treatment or starvation in HeLa cells and cardiomyocytes, autosis has been observed in more physiological in vivo models including in kidneys of mice after renal ischemia/reperfusion, in the hearts and livers of starved mice and in the heart after ischemia/reperfusion [16, 18]. All the morphological features of autosis were also observed in dying neurons after rat perinatal cerebral hypoxia-ischemia (HI) [12, 15] and the cardiac glycoside neriifolin (Nerii) was shown to be highly neuroprotective in these models of perinatal asphyxia [12, 20].

Over the past decade, we and others have participated to demonstrate that inhibiting HI-enhanced autophagy decreased neuronal death mechanisms leading to cerebral lesions in different rodent models of perinatal hypoxic/ischemic brain injuries [12, 21–26]. Treatment with 3-methyladenine, a pharmacological inhibitor of PI3KC3, strongly reduced brain lesions induced by middle cerebral artery occlusion [24] or by intracerebral injection of the glutamate analog ibotenate [21] in rat pups. More relevant, knockdown (KD) or knockout of the autophagic genes *Becn1* or *Atg7* decreased brain damage in rodent models of perinatal asphyxia [23, 25, 26]. Since we reported that dying neurons in the brain of human newborns with severe hypoxic-ischemic encephalopathy (HIE) were highly autophagic [15, 25], we can speculate that brain damage is also involving autosis in neurons of human asphyxiated newborns. A better understanding of neuronal autosis involvement and mechanisms could then allow to pave the way for developing new neuroprotective strategies not only in the context of HIE but also in other neuronal death conditions related to deleterious autophagy.

In order to investigate the relevance and some downstream mechanisms of autosis in neuronal death in hypoxic-ischemic conditions, the present study used in vitro and in vivo rat models of HI-mediated neuronal death and autoptic human brains of newborns with severe HIE.

## Results

### Neurotoxic concentrations of the autophagy-inducing peptide Tat-BECN1 trigger autosis in primary cortical neurons

**Fig. 1:**
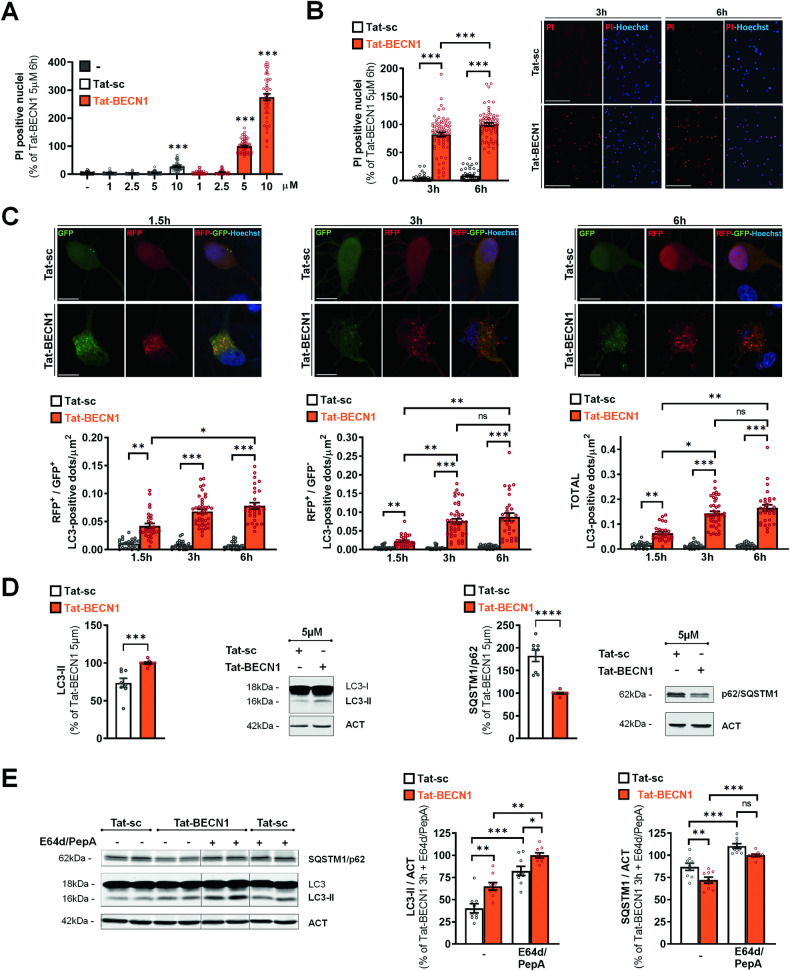
High concentrations of Tat-BECN1 induce neuronal death and increase autophagy flux in primary cortical neurons. **A** Tat-BECN1 increases the percentage of propidium iodide (PI)-positive nuclei at both 5 and 10 µM after 6 h of treatment compared to non-treated neurons. Tat-sc has a slight toxic effect from 10 µM. Dunn’s multiple comparisons test, compared to non-treated neurons. n ≥ 36 per condition. 4 independent experiments. **B** The percentage of PI-positive nuclei increases from 3 h in the presence of 5 µM Tat-BECN1 as illustrated in representative images of PI stainings. n ≥ 48 per condition. Tukey’s multiple comparisons test. 4 independent experiments. ***p < 0.001. Scale bar = 100 µm. **C** Representative confocal microscopy images of primary cortical neurons transfected with the RFP-GFP-LC3 plasmid in Tat-sc and Tat-BECN1 (5 μM) conditions at 1.5 h, 3 h and 6 h. Scale bar = 10 µm. The number of both GFP^+^ - RFP^+^ (yellow) and RFP^+^-GFP^−^ (red) -LC3-positive dots and the total number of LC3-positive dots significantly increase at all time points with Tat-BECN1 treatment. Dunn’s multiple comparisons test, n ≥ 25 per condition; 5 independent experiments. **D** Representative immunoblots and corresponding quantifications of LC3-II (Unpaired t-test) and SQSTM1/p62 (Mann–Whitney test) after 6 h of Tat-BECN1 (5 μM) treatment (n ≥ 8 per condition, 4 independent experiments) and **E** after 3 h with or without the lysosomal enzymes inhibitors cocktail E64d/PepstatinA (E64d/PepA) treatment. Both SQSTM1/p62 and LC3-II expression levels are significantly increased by E64d/PepA in basal and Tat-BECN1 conditions. n ≥ 9 per condition. Dunn’s multiple comparisons test. 3 independent experiments. Values are mean ± SEM. *p < 0.05, **p < 0.01, ***p < 0.001; ns not significant.

To determine the neurotoxicity of Tat-BECN1, we first used logarithmic concentrations of Tat-BECN1 and Tat-sc in primary cortical neuronal cultures (Supplementary Fig. S1A, B). We showed that Tat-BECN1 induces neuronal death at concentrations starting from 10 μM, as detected with either PI staining (Supplementary Fig, S1A) or lactate dehydrogenase (LDH) released assay (Supplementary Fig, S1B). However, since the control peptide, Tat-sc (scrambled sequence of Tat-BECN1) was slightly but significantly neurotoxic from 10 µM, we analyzed the effect of arithmetic concentrations (1–10 μM) of Tat-BECN1. We then determine that 5 μM of Tat-BECN1 is the most appropriate neurotoxic concentration for our study in primary cortical neuronal cultures, as shown by a significant increase in PI-positive nuclei and LDH released at 6 h (Fig. 1A and Fig. S1C). The concentration of 5 µM of Tat-BECN1 was then used to further characterize Tat-BECN1-induced neuronal death. Cell death was detected as soon as 3 h after Tat-BECN1 addition (Fig. 1B).

We then evaluated whether Tat-BECN1-induced neurotoxicity was associated to increased autophagy flux in primary cortical neuronal cultures. Using a plasmid expressing rat LC3 fused to mRFP and eGFP (pftLC3) [27], we showed that Tat-BECN1 treatment significantly increased the numbers of both RFP^+^-GFP^+^ (early autophagosomes; yellow) and RFP^+^-GFP^−^ (late autophagosomes; red) dots at 1.5 h, 3 h and 6 h after Tat-BECN1 treatment compared to control conditions (Tat-sc), indicating a progressive increase in the autophagy flux (Fig. 1C). Western blot analyses showed that Tat-BECN1-induced increase in LC3-II levels occurred in parallel with a decreased expression of SQSTM1/p62 confirming an enhanced autophagic flux [28, 29] at 6 h after Tat-BECN1 treatment (Fig. 1D). Moreover, LC3-II levels were significantly higher when lysosomal degradation was efficiently blocked by the inhibitor cocktail of E64d/Pepstatin A [21, 23] in the presence of Tat-BECN1 (5 µM) than in control conditions confirming that Tat-BECN1 induced the formation of new autophagosomes (Fig. 1E). We can note that lower, non-neurotoxic, concentration of Tat-BECN1 (2.5 µM) was also able to enhance autophagy flux, but to a lesser extent (Fig. S1).

**Fig. 2:**
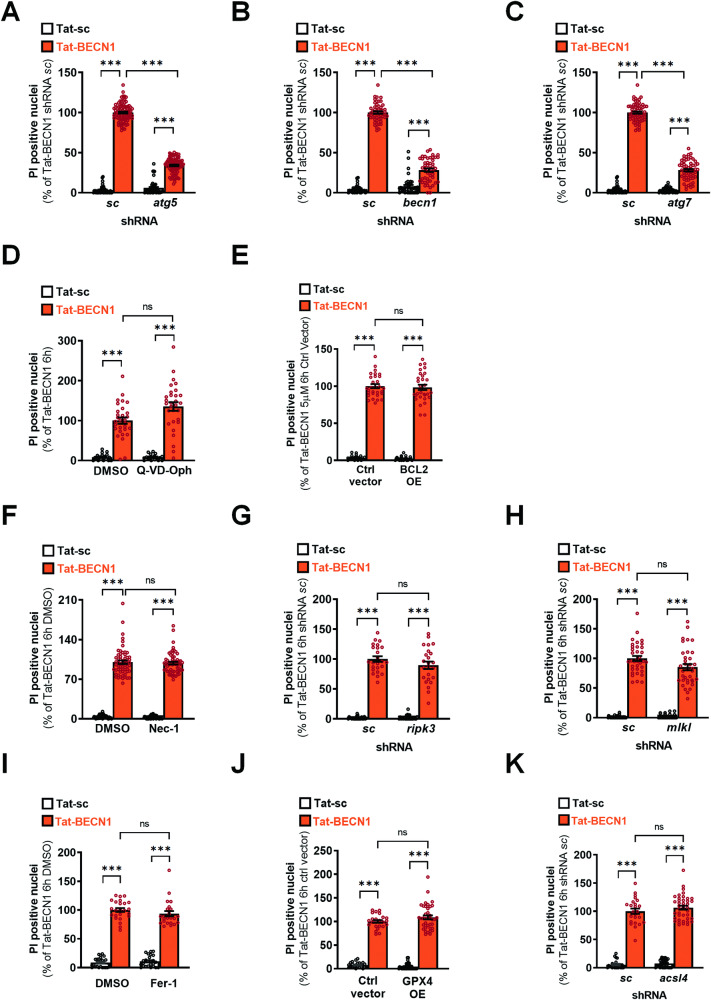
Tat-BECN1-induced neuronal death in primary cortical neurons is autophagy-dependent and is not involving apoptosis, necroptosis and ferroptosis. Downregulation of (**A**) ATG5 (n ≥ 47 per condition, 5 independent experiments), (**B**) BECN1 (n ≥ 36 per condition, 3 independent experiments) and (**C**) ATG7 (n ≥ 42 per condition, 4 independent experiments) reduces neuronal death as shown by a decrease in the percentage of propidium iodide (PI) positive nuclei. Dunn’s multiple comparisons test. **D** The pharmacological pan-caspase inhibitor Q-VD-Oph (25 µM) does not reduce the percentage of PI positive nuclei after 6 h of Tat-BECN1 treatment (n ≥ 30 per condition). **E** BCL2 overexpression (BCL2 OE) has no effect on Tat-BECN1 increased percentage of PI-positive nuclei after Tat-BECN1 treatment (n ≥ 24 per condition). **F** Pharmacological inhibition of necroptosis with Necrostatin 1 (Nec-1, 30 µM) does not reduce Tat-BECN1-induced increase percentage of PI-positive nuclei (n ≥ 46 per condition). **G** Lentiviral-mediated downregulation of RIPK3 (n ≥ 24 per condition) or **H** of MLKL (n ≥ 36 per condition) has no effect on the percentage of PI-positive nuclei. **I** Pharmacological inhibition of ferroptosis with Ferrostatin-1 (Fer-1, 10 µM), does not reduce the percentage of PI-positive nuclei (n ≥ 24 per condition; Dunn’s multiple comparisons test, 3 independent experiments). **J** Lentiviral-mediated overexpression of GPX4 (GPX4 OE) (n ≥ 24 per condition) or **K** knockdown of ACSL4 (n ≥ 24 per condition) have not effect on PI-positive nuclei number. Dunn’s multiple comparisons test, 3 independent experiments. Values are mean ± SEM. ***p < 0.001; ns: not significant.

Then, we investigated whether Tat-BECN1-induced neuronal death is autophagy-dependent using lentiviral transduction of shRNAs to efficiently downregulate the expression of three different important autophagy-related (ATG) proteins involved in autophagosome formation (ATG7, ATG5 and BECN1). We observed that a KD efficiency of more than 60% for the different ATGs (Fig. S2A–C), strongly reduced Tat-BECN1-induced autophagy flux monitored with the RFP-GFP-LC3 plasmid (Fig. S2D, E) and significantly decreased Tat-BECN1-induced neuronal death by more than 70% (Fig. 2A–C).

The next step consisted in demonstrating that Tat-BECN1-induced neuronal death was not dependent on another regulated cell death pathway. Inhibition of apoptotic mechanisms, either with the pharmacological pan-caspase inhibitor Q-VD-Oph (25 µM) (Fig. S2F) or with the overexpression of BCL-2 (BCL2-OE) (Fig. S2G, H), efficiently prevented CASPASE-3 cleavage induced by a classical apoptotic stimulus, staurosporin (STS) [30]. However, both anti-apoptotic strategies had no effect on Tat-BECN1-induced neuronal death (Fig. 2D, E). To evaluate the involvement of necroptosis in Tat-BECN1-induced neuronal death we used a pharmacological inhibitor, Necrostatin-1 (30 µM) (Fig. S2I) [31–33] and the downregulation of two key proteins involved in necroptosis signaling, MLKL (Fig. S2J) and RIPK3 (Fig. S2K). These strategies were efficient in preventing neuronal death triggered by shikonin (20 µM), a well-characterized pro-necroptotic stimulus [34, 35] (Fig. S2I–K), but had no effect on Tat-BECN1-induced neuronal death (Fig. 2F–H). Then to exclude the involvement of ferroptosis in Tat-BECN1-induced neuronal death, we used either a pharmacological inhibitor, Ferrostatin-1 [36] (Fer-1, 10 µM), or genetical overexpression of Glutathione peroxidase 4 (GPX4) [37], a main regulator of ferroptosis, or downregulation of acyl-CoA synthetase long-chain family member 4 (ACSL4) [38]. Despite we showed that both strategies were neuroprotective in the pro-ferroptotic conditions RSL3 [39] (20 µM, 24 h) treatment (Fig. S2L–N), inhibiting ferroptosis was not efficient to significantly decrease Tat-BECN1-induced neuronal death (Fig. 2I–K).

**Fig. 3:**
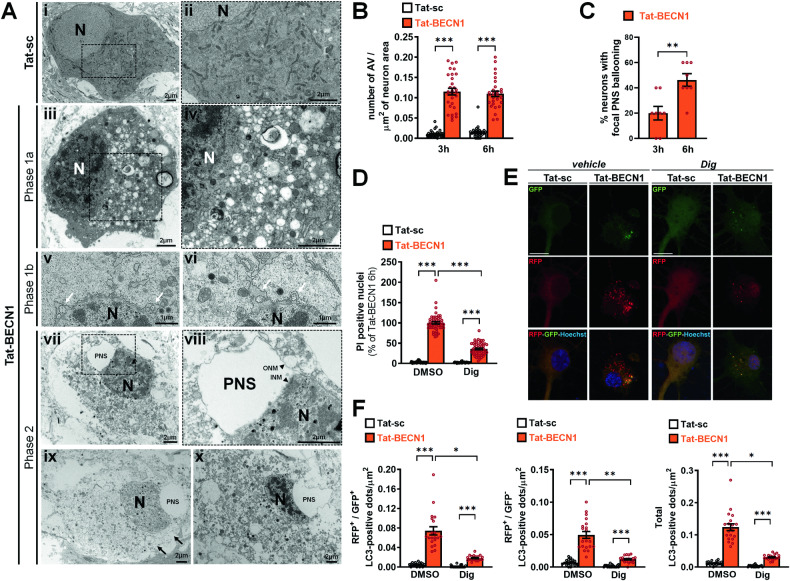
Tat-BECN1-induced neuronal death has morphological and biochemical features of autosis. **A** Representative electron micrographs of primary cortical neurons treated with Tat-scrambled (Tat-sc) or Tat–BECN1 for 3 h. Tat-BECN1 treatment induces typical morphological features of (**iii–iv**) phase 1a autosis including presence of convoluted nuclei accompanied by endoplasmic reticulum dilatation and fragmentation, and of numerous autophagosomes, autolysosomes and empty vesicles; (**v–vi**) phase 1b autosis with restricted swollen perinuclear space (PNS) regions (white arrows) containing lumps of cytosol and (**vii–x**) phase 2 autosis, as shown for different dying neurons, with the presence of a focal ballooning of the (empty) perinuclear space (PNS) associated with a concavity of the nucleus, a swelling of almost all organelles and loss of plasma membrane integrity (black arrows). These morphological features are not observed in Tat-sc treated neurons (**i**, **ii**). N: nucleus, ONM: outer nuclear membrane; INM: inner nuclear membrane; PNS, perinuclear space. **B** EM quantifications showed that the number of autophagic vacuoles (AV) per neuron increases after both 3 h and 6 h of Tat-BECN1 treatment compared to Tat-sc condition (n ≥ 30, Dunn’s multiple comparisons test; 3 independent experiments). **C** The percentage of neurons with a focal ballooning of the PNS (not detected in Tat-sc condition) significantly increases between 3 h and 6 h of Tat-BECN1 treatment. (n ≥ 80 neurons per condition; Unpaired t-test; 3 independent experiments). **D** Tat-BECN1-induced neuronal death is sensitive to cardiac glycosides. The percentage of propidium iodide (PI) positive nuclei is also significantly decreased by digoxin (dig, 50 µM) treatment at 6 h. n ≥ 25 per condition, Dunn’s multiple comparison test; 3 independent experiments). **E** Representative confocal microscopy images of RFP-GFP-LC3 transfected primary cortical neurons and **F** corresponding quantifications showing the effect of dig on Tat-BECN1-induced autophagy flux. The number of RFP^+^-GFP^+^-LC3-positive dots (yellow), of RFP^+^-GFP^-^-LC3-positive dots (red) and of the total LC3-positive dots are significantly reduced in the presence dig after 3 h of Tat-BECN1 treatment. Dunn’s multiple comparison test, n ≥ 20 per condition, 3 independent experiments. Scale bar = 10 µm. *p < 0.05, **p < 0.01, ***p < 0.001.

The last step was to demonstrate that Tat-BECN1-mediated neuronal death showed all the morphological and biochemical features of autosis. Following Tat-BECN1 treatment, dying neurons with morphological characteristics of the different phases of autosis were observed by electron microscopy (EM). As shown in Fig. 3A iii–iv, some dying neurons presented morphological features of phase 1a with convoluted nuclei (not associated with classical chromatin condensation and without apparent changes in the perinuclear space (PNS)), some electron dense mitochondria, dilated and fragmented endoplasmic reticulum (ER), and the cytosol filled by numerous autophagosomes, autolysosomes and empty vacuoles. EM quantification of the number of autophagic vacuoles (AV) confirmed that Tat-BECN1 treatment increased the number of AV at both 3 h and 6 h after Tat-BECN1 treatment (Fig. 3B). Phase1b morphological features were also detected in some dying neurons consisting in discrete and restricted swollen PNS regions containing cytosolic-like clumps (Fig. 3A v–vi). We also observed dying neurons displaying phase 2 autosis morphological criteria characterized by a necrotic-like morphology (swelling of almost all organelles, loss of plasma membrane integrity) and the main morphological signature of autosis, i.e. a focal ballooning of the empty PNS associated with a nucleus concavity (Fig. 3Avii–x) that was not detected in Tat-sc treated neurons (Fig. 3Ai–ii). EM quantification of the number of dying neurons with this typical focal swelling of the PNS showed that Tat-BECN1 treatment induced a progressive increase in the occurrence of this morphological signature from 3 to 6 h after Tat-BECN1 treatment (Fig. 3C).

In addition to its specific morphological features, autosis has been biochemically characterized by its dependency on ATP1a and thus its sensitivity to cardiac glycosides [12]. Treatment with the cardiac glycoside digoxin (Dig) (50 nM) significantly reduced Tat-BECN1-induced neuronal death by 65% (Fig. 3D). Moreover, confocal microscopy analyses of RFP-GFP LC3 transfected neurons confirmed that Dig treatment strongly reduced Tat-BECN1-induced autophagy flux (Fig. 3E, F).

Altogether, these results showed that Tat-BECN1-induced neuronal death has all the morphological and biochemical features of autosis.

### Tat-BECN1-induced neuronal autosis is dependent on alpha 3 subunit of Na^+^/K^+^-ATPase

**Fig. 4:**
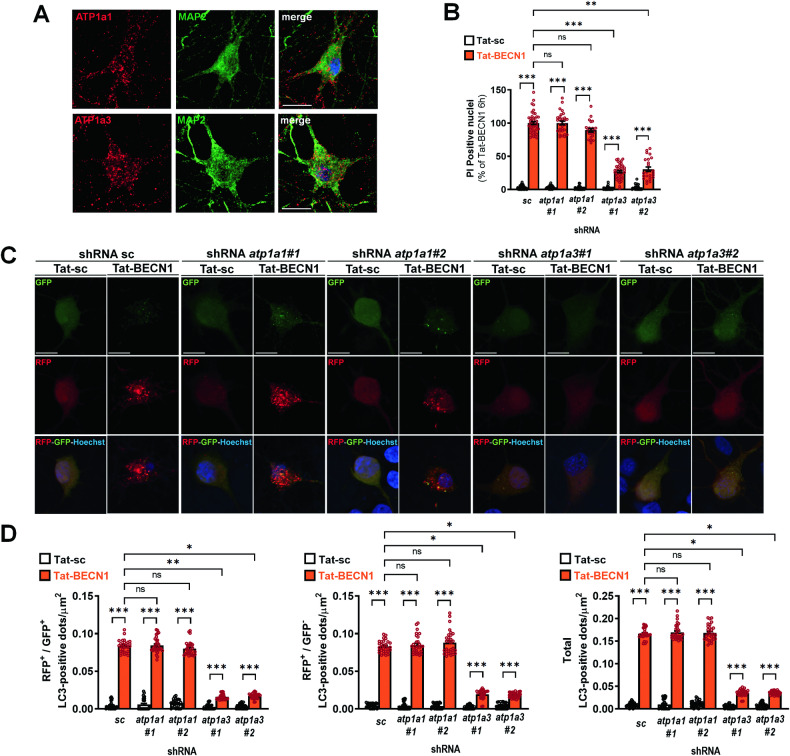
Tat-BECN1-induced neuronal autosis is dependent on ATP1a3. **A** Representative confocal microscopy images of ATP1a1 and ATP1a3 (in red) in primary cortical neurons (MAP2, in green). Scale bar = 10 µm. **B** Downregulation of ATP1a1 (with both shRNA *atp1a1#1* and shRNA *atp1a1#2*) has no effect on the percentage of propidium iodide (PI)-positive nuclei induced by 6 h of Tat-BECN1 treatment whereas downregulation of ATP1a3 (with both shRNA *atp1a3#1* and shRNA *atp1a3#2)* significantly reduces the number of PI-positive nuclei (n ≥ 24 per condition; Dunn’s multiple comparisons test; 3 independent experiments). **C** Representative confocal microscopy images of RFP-GFP-LC3 transfected primary cortical neurons of Tat-sc and Tat-BECN1-treated neurons transduced with shRNAs *atp1a1#1, atp1a1#2, atp1a3#1* and *atp1a3#2*. Scale bar = 10 µm. **D** Downregulation of ATP1a1 has no significant effect 3 h after Tat-BECN1 treatment on the number of RFP^+^-GFP^+^LC3 positive dots (yellow, early autophagosomes), of RFP^+^-GFP^−^-LC3 positive dots (red, late autophagosomes) and on the total number of positive dots. In Tat-BECN1 conditions, downregulation of ATP1a3 significantly decreases the number of RFP^+^-GFP^+^-LC3 positive dots, the number of RFP^+^-GFP^-^LC3 positive dots and the total number of LC3-positive dots (n ≥ 30 per condition, Dunn’s multiple comparison test, 3 independent experiments). Values are mean ± SEM. *p < 0.05, **p < 0.01, ***p < 0.001, ns: not significant.

Primary cortical neurons express both ATP1a1 and ATP1a3 [40] as shown by immunocytochemistry (Fig. 4A), immunoblots (Fig. S3) and qPCR analyses (Fig. S3H). However, qPCR analyses revealed that ATP1a1 mRNA are 10-fold less expressed than ATP1a3 mRNA in our primary cortical neuronal cultures (Fig. S3H). Then, we evaluate the contribution of ATP1a1 and ATP1a3 subunits in neuronal autosis. Downregulation of ATP1a1 (Fig. S3A, B) had no effect against Tat-BECN1-induced neuronal death (Fig. 4B) and enhanced autophagy (Fig. 4C, D). On the contrary, ATP1a3 downregulation (Fig. S3C, D) afforded neuroprotection (by approximately 75%) (Fig. 4B), and strongly reduced the autophagy flux (Fig. 4C, D). By immunoblots we showed that downregulation of ATP1a1 did not affect the expression levels of ATP1a3 (Fig. S3E, F) and ATP1a1/ATP1a3 double KD experiments showed a similar neuroprotective effect than ATP1a3 KD alone (Fig. S3G), indicating that ATP1a1 did not, even partially, participate in Tat-BECN1-induced neuronal death.

### Hypoxic-excitotoxic neuronal death involves ATP1a3-dependent autosis in primary cortical neurons

We previously showed that a transient exposure (30 min) to kainate (Ka, 30 µM) combined with hypoxia (Hx, 6% oxygen) induced neuronal death in primary cortical neurons which was decreased by downregulation of BECN1 and ATG7 and exacerbated by the overexpression of these two ATG proteins [23]. Similarly to Tat-BECN1, KaHx induced a neuronal death independent of apoptosis (not sensitive to BCL2 overexpression or to Q-VD-Oph treatment) [23], of necroptosis (not sensitive to Necrostatin-1 (Fig. S4B), MLKL KD (Fig. S4C) and RIPK3 KD (Fig. S4A)) and of ferroptosis (not sensitive to Fer-1 (Fig. S4D), GPX4 overexpression (Fig. S4E) and to ACSL4 KD (Fig. S4F)).

**Fig. 5:**
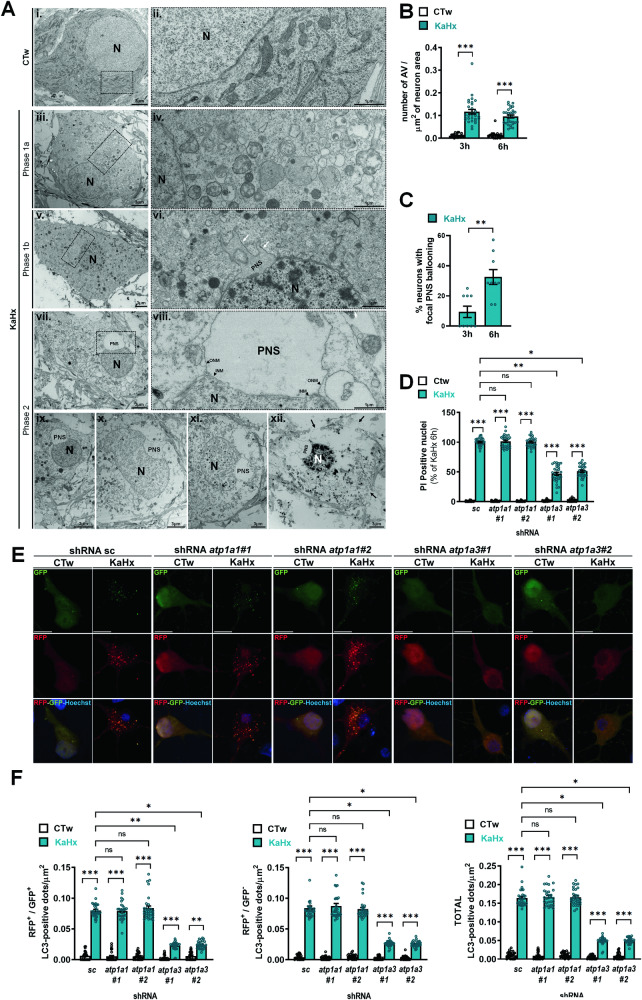
Kainate-Hypoxia-induced neuronal death involves ATP1a3-dependent autosis in primary cortical neurons. **A** Representative electron micrographs of primary cortical neurons 3 h after CTw or kainate-hypoxia (KaHx) treatment. KaHx-treated primary cortical neuronal cultures showed some dying neurons with morphological characteristics of (**iii-iv**) phase 1a autosis such as dilatation and fragmentation of endoplasmic reticulum; presence of numerous autophagosomes, autolysosomes and empty vacuoles in the cytosol; (**v–vi**) phase 1b autosis with restricted swollen perinulear space (PNS) regions (white arrows) containing clumps of cytoplasmic material; and (**vii–xii**) phase 2 autosis, as shown for different dying neurons, with the presence of a focal ballooning of the (empty) perinuclear space (PNS) associated with a concavity of the nucleus, a swelling of almost all organelles and loss of plasma membrane integrity (black arrows). These morphological features are not observed in CTw-treated neurons (**i**, **ii**). N: nucleus, ONM: outer nuclear membrane; INM: inner nuclear membrane; PNS, perinuclear space. **B** The number of autophagic vacuoles (AV) per neuron increases at both 3 h and 6 h after KaHx treatment compared to CTw condition (n ≥ 30; Dunn’s multiple comparisons test; 3 independent experiments). **C** The percentage of dying neurons with a focal ballooning of the PNS (not observed in CTw condition) significantly increases between 3 h and 6 h after KaHx treatment (n ≥ 90 neurons per condition; Mann-Whitney test; 3 independent experiments). **D** Downregulation of ATP1a1 (with both shRNA *atp1a1#1* and shRNA *atp1a1#2*) has no effect on the percentage of propidium iodide (PI)-positive nuclei induced by 6 h of KaHx whereas downregulation of ATP1a3 (with both shRNA *atp1a3 #*and shRNA *atp1a3 #2*) significantly reduces the number of PI-positive nuclei (n ≥ 42 per condition; Dunn’s multiple comparisons test; 3 independent experiments). **E** Representative confocal microscopy images of RFP-GFP-LC3 transfected primary cortical neurons transduced with shRNAs *atp1a1#1, atp1a1#2, atp1a3#1* and *atp1a3#2* after CTw and KaHx treatment. Scale bar = 10 µm. **F** Downregulation of ATP1a1 has no significant effect on the RFP-GFP LC3-positive dots pattern 3 h after KaHx treatment. In KaHx conditions, downregulation of ATP1a3 significantly decreases the number of RFP^+^-GFP^+^LC3-positive dots (yellow, early autophagosomes), the number of RFP^+^-GFP^-^LC3-positive dots (red, late autophagosomes) and the total number of LC3-positive dots (n ≥ 30 per condition, Dunn’s multiple comparison test, 3 independent experiments). Values are mean ± SEM. *p < 0.05, **p < 0.01, ***p < 0.001, ns not significant.

To evaluate whether autosis could be involved in KaHx-induced neuronal death, we then investigated the ultrastructural features of KaHx-treated primary cortical neurons by EM. Some dying neurons displayed morphological features of the different phases of autosis: phase 1a (presence of numerous autophagic vacuoles and empty vesicles in the cytosol (Fig. 5Aiii–iv)), phase 1b (restricted swelling of a region of the PNS containing clumps of cytosol (Fig. 5Av–vi)) and phase 2 (swelled organelles and rupture of the plasma membrane, and a focal ballooning of the PNS associated with a concavity of the nucleus (Fig. 5Avii–xii)). EM quantification of the number of AV confirmed the KaHx-induced strong increase in the number of AV at both 3 h and 6 h after the insult (Fig. 5B). Quantification of the number of dying neurons with a focal ballooning of the PNS showed a progressive increase in neurons with this typical morphological signature between 3 h and 6 h after KaHx treatment (Fig. 5C), whereas they were not observed in control condition (CTw) (Fig. 5Ai–ii).

Finally, KaHx-induced neuronal death (Fig. 5D) and autophagy (Fig. 5E, F) were significantly reduced by ATP1a3 KD (and not affected by ATP1a1 KD) demonstrating that neuronal ATP1a3-dependent autosis is involved in hypoxic-ischemic conditions in primary cortical neurons.

### Increased ATP1a3-BECN1 interaction in in vitro and in vivo rat models of neuronal autosis and in dying neurons of human newborns with hypoxic-ischemic encephalopathy

**Fig. 6:**
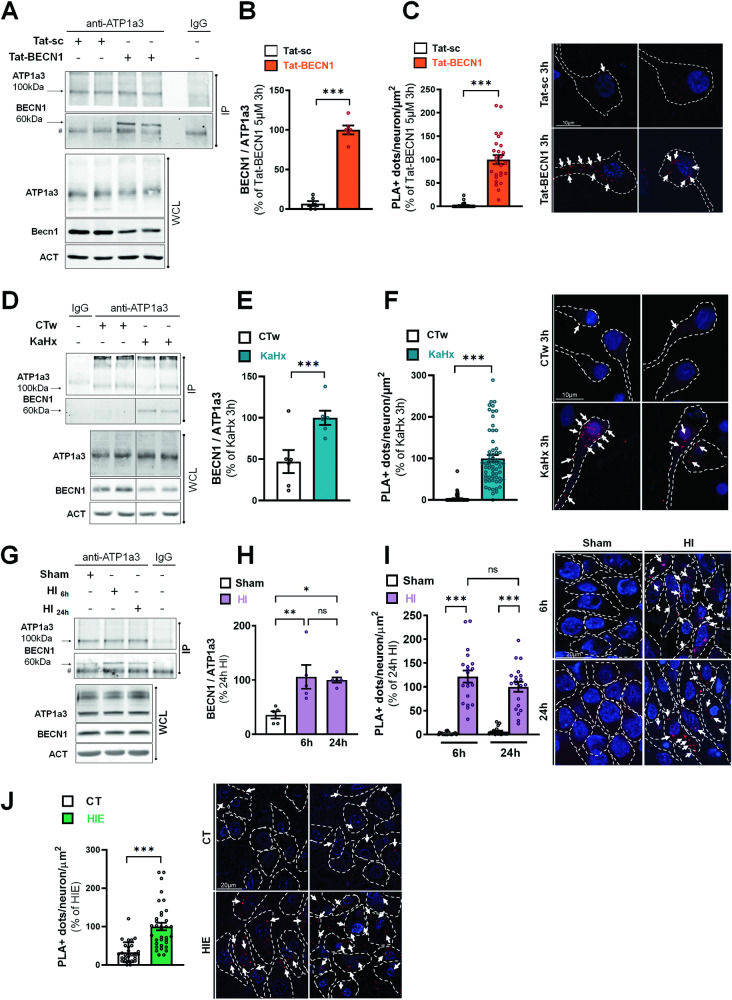
Increased BECN1-ATP1a3 interaction in in vitro and in vivo rat models of neuronal autosis and in dying neurons of human newborns with hypoxic-ischemic encephalopathy. **A** Representative immunoblots and **B** corresponding quantifications of coimmunoprecipitation of BECLIN1 (BECN1) with ATP1a3 in primary cortical neurons treated with Tat-BECN1 or Tat-sc for 3 h (n = 6, unpaired t-test; 4 independent experiments). **C** Representative images and corresponding quantifications of proximity ligation assays (PLAs) of BECN1 and ATP1a3 in primary cortical neurons treated with Tat-BECN1 or Tat-sc for 3 h (n ≥ 25, Mann Whitney test; 3 independent experiments; scale bar = 10 µm). **D** Representative immunoblots and **E** corresponding quantifications of coimmunoprecipitation of BECN1 with ATP1a3 in primary cortical neurons treated with kainate-hypoxia (KaHx) or control CTw for 3 h (n = 6, unpaired t-test; 3 independent experiments). **F** Representative images and corresponding quantifications of PLAs of BECN1 and ATP1a3 in primary cortical neurons treated with kainate-hypoxia (KaHx) or control CTw for 3 h (n ≥ 60, Mann Whitney test; 2 independent experiments; scale bar = 10 µm). **G** Representative immunoblots and **H** corresponding quantifications of coimmunoprecipitation of BECN1 with ATP1a3 in hippocampus samples of rat pups after 6 h and 24 h of hypoxia-ischemia (HI) and in corresponding sham animals (n = 5 animals/condition, Tukey’s multiple comparison test; 4 independent experiments). **I** Representative images and corresponding quantifications of PLAs of BECN1 and ATP1a3 in CA3 hippocampus region of rat pups after 6 h and 24 h of hypoxia-ischemia (HI) and in corresponding sham animals (n = 20 images analyzed from 4 animals/condition, Dunn’s multiple comparison test; 3 independent experiments; scale bar = 20 µm). **J** Representative images and corresponding quantifications of PLAs of BECN1 and ATP1a3 in CA3 hippocampus region of human control (n = 5 cases) and hypoxic-ischemic-encephalopathy (HIE; n = 5 cases) newborns (n ≥ 5 images analyzed / case, Mann Whitney test; scale bar = 20 µm). Values are mean ± SEM. *p < 0.05, **p < 0.01, ***p < 0.001, ns: not significant.

Because we identified ATP1a3 as the main ATP1a subunit mediating neuronal autosis and since we previously demonstrated that ATP1a and BECN1 interacted in different cell types in autosis conditions [16], we then investigated whether BECN1 specifically interacts with ATP1a3 in neuronal autosis. We found that ATP1a3-BECN1 interaction occurred in primary cortical neurons following both Tat-BECN1 (Fig. 6A–C) and KaHx treatments (Fig. 6D–F). For both stimulus, immunoprecipitation of ATP1a3 followed by immunoblot of BECN1 showed a significant increase of BECN1 binding to ATP1a3 (Fig. 6A, B, D, E). ATP1a3-BECN1 interaction was confirmed by an increased number of PLA positive dots in Tat-BECN1 (Fig. 6C) and KaHx (Fig. 6F) compared to control conditions. Moreover, this interaction was strongly reduced by Dig treatment (Fig. S5A). Of note, we were unable to immunoprecipitate BECN1 with ATP1a1 (Fig. S5B) suggesting that BECN1 could specifically interact with ATP1a3 to mediate neuronal autosis.

ATP1a3-BECN1 interaction was also found to occur in vivo in hypoxic-ischemic rat pups at both 6 and 24 h after the insult as shown by both immunoprecipitation (Fig. 6G, H) and PLA (Fig. 6I). Similarly, to in vitro autosis conditions, this interaction was strongly reduced when rat pups received an intraperitoneal injection of the cardiac glycoside Nerii (0.22 mg/kg) just before hypoxia (Fig. S5C, D).

We then investigated the presence of ATP1a3-BECN1 interaction in autoptic brain sections from human term newborns presenting severe HIE and dying neurons with a high autophagic features [15, 25]. A strong increase in PLA positive dots was detected in dying neurons of HIE human term newborns compared to control cases (Fig. 6J) suggesting that ATP1a3-BECN1-dependent neuronal autosis is also involved in neuronal death mechanisms in severe HIE.

## Discussion

Autosis has been mainly described in dividing cell types (HeLa, MEFs, cardiomyocytes…) over the past years [12, 18] and we reported the presence of autosis morphological features in dying neurons after perinatal cerebral HI in rat pups [12, 15]. Since we previously provided evidence for a pro-death role of enhanced autophagy mechanisms in different rodent models of acute perinatal brain injuries [12, 15, 21–25], the present study aimed to investigate the involvement and the relevance of autosis in neurons, long-lived post-mitotic cells for which autophagy is of great importance to keep cell homeostasis and quality control.

Since focal plasma membrane rupture occurs once the focal ballooning of the PNS is formed [12] at the latest stage of autosis (phase 2), we used PI staining to evaluate cell death mediated by autosis. Cell death was also assessed by measuring the LDH release in the medium and similar results were obtained in all conditions tested (not shown). Autosis was first described in HeLa cells treated with a high concentration of Tat-BECN1 [12], a peptide known to activate PI3KC3 complexes (C1 and C2) [41, 42] and thus boosting the autophagic flux. Despite this peptide has been recommended for inducing autophagy as a therapeutic strategy for pathologies such as Huntington’s disease or cancer [13], Tat-BECN1 could also promote toxicity (autosis) in a concentration dependent manner that is cell type specific. Whereas toxicity is reached in HeLa cells between 10 and 20 µM [12, 13], 2.5 µM of Tat-BECN1 is sufficient to affect cardiomyocytes survival [18]. Finding the appropriate concentration to induce neuronal death was thus the first step for our study. Autophagic flux was progressively increased by Tat-BECN1 from 2.5 µM (as shown by SQSTM1/p62 reduction) in primary cortical neurons but significant toxicity started from 5 µM suggesting that autosis could be induced when autophagic flux is above a critical threshold.

Of interest, we here also provided evidence that neurons could undergo autosis not only when treated with Tat-BECN1, but also after a hypoxic-ischemic stimulus (mimicking neuronal death induced by cerebral HI) previously shown to highly activate deleterious autophagy (KaHx) [23]. The presence of autophagic cell death features in both in vitro [23] and in vivo [12, 15] rat models of perinatal cerebral HI indicates that neuronal autosis should be considered as an important mechanism of neuronal death occurring in HI conditions.

The debate surrounding the role of autophagy in cell death (die by autophagy vs with autophagy) has been partially mitigated by an update classification of cell death types focused on molecular features rather than on morphological aspects since morphological presence of enhanced autophagy is insufficient to prove that autophagy can kill [11]. The Nomenclature Committee on Cell Death (NCCD) has determined specific criteria for autophagic cell death and described it as “a form of Regulated Cell Death that mechanistically depends on autophagic machinery” [10], meaning that autophagic cell death depends only on autophagic genes and not on other cell death molecular pathways such as those conducting apoptosis or necroptosis [10, 11, 43]. We here showed that neuronal autosis is, as recommended by the NCCD, dependent on canonical autophagy machinery (ATG5, ATG7 and BECN1) and independent of apoptosis and necroptosis pathways. Moreover, in this study, we provide a step further, since several studies suggested that autophagy could regulate ferroptosis [44, 45], we demonstrate that, in the pro-autosis conditions tested (Tat-BECN1 and KaHx treatments), ferroptosis is also not involved in neuronal death. Altogether, we provided evidence that neuronal autosis is independent of other RCD pathways (apoptosis, necroptosis and ferroptosis), confirming that autosis is a form of autophagic cell death.

Autosis has been molecularly characterized by its dependency on ATP1a1 in many different dividing cell types (HeLa, MEFs, BMDM, MDM, cardiomyocytes, U2OS) [12, 13, 18, 46]. In mammals, four different catalytic ATP1a subunits exist [47]. Whereas ATP1a1 is ubiquitously expressed, ATP1a3 is predominantly expressed in the brain and specifically by neurons [40, 48–50]. Interestingly, autosis-mediated neuronal death was only sensitive to ATP1a3 KD (not to ATP1a1 KD) showing that neuronal autosis is specifically mediated by ATP1a3.

It can be noted that human neurological syndromes are to date only related to ATP1a3. There is growing evidence that ATP1a3 mutations are involved in many pediatric neurological diseases such as alternating hemiplegia, sensorineural hearing loss syndrome or again rapid-onset dystonia-Parkinson and early infantile epileptic encephalopathy [49–52]. In human adult brain, ATP1a3 appeared also to be defective in Alzheimer’s disease, a brain pathology involving impaired autophagy as others proteinopathies [52–56]. The age-related ATP1a3 mRNA reduction seemed to be faster in Alzheimer’s disease patients compared to control aged-patient and precedes diffuse plaque apparition [57]. More recent studies revealed that the amyloid-β plaques could bind to ATP1a3 and block its activity, suggesting that this interaction is partly responsible of neuronal degeneration in Alzheimer’s disease [56, 58]. On the contrary of ATP1a3, ATP1a1 expression did not seem to be affected in Alzheimer’s disease patients [56]. Concerning Parkinson’s Disease, it has been demonstrated that α-synucleins clusters could interact with ATP1a3, and their accumulation decreases ATP1a3 efficiency [52, 59]. Here again, ATP1a1 was not affected by α-synucleins aggregates [52, 59]. These results suggest then a strong link between ATP1a3 and neuronal autophagy.

Because ATP1a3 has a lower Na^+^ affinity, it has been then proposed that ATP1a3 is mainly activated during rise in intracellular Na^+^ concentration with the crucial role to restore intracellular Na^+^ level through a rapid Na^+^ extrusion [60, 61]. Further investigation is required to investigate the role of intracellular Na^+^ levels after Tat-BECN1 treatment, but excitotoxic stimuli, such as KaHx or HI, are characterized by both Na^+^ and Ca^2+^ massive influxes. We can then postulate that the ATP1a3-dependency of neuronal autosis could be related to the lower Na^+^ affinity of this subunit.

Moreover, we showed that Dig could be efficient to decrease autosis at nanomolar concentrations in neurons reflecting certainly the higher affinity for ATP1a3 than ATP1a1 of these cardiac glycosides [62–64]. Very low concentrations of cardiac glycosides which do not change intracellular ion concentration have been shown to affect numerous multiple cells signaling pathways (growth, differentiation, proliferation, apoptosis…) [63, 65, 66]. These effects led to the discovery that ATP1 could act, independently of its ion pump properties, as a scaffold protein forming signaling modules (as a signal transducer) resulting in cascades of protein tyrosine phosphorylation and in second messenger production [67–70]. However, most of the studies on the signal transducer function of ATP1 focused on ATP1a1 in cell lines. To support this hypothesis, we here provided evidence that neuronal autosis is associated with an increase in ATP1a3-BECN1 interaction and that neuroprotection with cardiac glycosides is associated with a decrease in this interaction in both in vitro and in vivo rat models of neuronal autosis. We can then propose that, in neurons, ATP1a3 could play a crucial role of signal transducer controlling Tat-BECN1 or KaHx-induced autophagy and autosis.

Cardiac glycosides have been previously identified as potent neuroprotectants in HI both in vitro and in vivo models [12, 20, 71, 72]. However, the narrow therapeutic range of concentrations of cardiac glycosides makes their use difficult at the clinical level [65, 73, 74], suggesting the need to find alternative neuroprotective strategy. Interestingly, we also demonstrated increased ATP1a3-BECN1 interaction in autoptic brains of human newborn with severe HIE, in which dying neurons displayed increased autophagy [15, 25]. Further investigation is now necessary to identify additional molecular signaling pathways specifically activated downstream of ATP1a3, but the important role of ATP1a3 in neuronal autophagy and autosis demonstrated in the present study paves the way for developing new neuroprotective strategies targeting ATP1a3-dependent autosis pathways specifically in neurons that could be potentially used in many neurological disorders including HIE.

## Materials and methods

### Primary cortical neuronal cultures

All animal experiments were performed according to the Swiss Laws for the protection of animals and were approved by the Vaud Cantonal Veterinary Office. Primary cortical neuronal cultures were done as described previously [75]. Pieces of the cortices of 1-day-old rat (Sprague Dawley; Janvier Labs, Saint Berthevin, France) were dissected and incubated for chemical dissociation with 200 units of papain (Sigma, #P3125-250MG, St. Louis, Missouri, USA) for 30 min at 34 °C. After a mechanical dissociation with a glass pipette, dissociated cortical neurons were plated at a density of 3.6·10^5^ cells/dish (35 mm poly-D-lysine precoated dishes, Corning, #356413, #356470, Corning, New York, USA) for biochemical analyses and LDH assays and at a lower density (2.5·10^5^ cells/ml) on 12 mm glass coverslips coated with poly-D-lysine (Sigma, #P4832 or Electronmicroscopy Sciences, #72294-02, Hartfield, Pennsylvania, USA) for immunocytochemistry and imaging analyses. For EM, primary cortical neurons were plated in poly-D-lysine (0.01%, Life Technologies, #A3890401, Carlsbad, California, USA)-coated glass slides (LabTek Chamber Slides, Thermo Fisher Scientific, #177399, Waltham, Massachusetts, USA) at a density of 2·10^5^ cells/ml. Primary cortical neurons were cultured in Neurobasal (Gibco/Thermo Fisher Scientific, #21103-049) supplemented with 2% B27 (Gibco/Thermo Fisher Scientific, #17504-044), 0.5 mmol/L glutamine (Sigma, #G8540-25G) and 100 mg/mL penicillin–streptomycin (Gibco/Thermo Fisher Scientific, #15140122) and maintained at 37 °C in a 5% CO_2_-containing atmosphere for at least 12 days. Half of the medium was changed every 3–4 days. Experiments were performed at 11–13 days in vitro (DIV).

### Rat model of perinatal asphyxia

All experiments were performed in accordance with Swiss laws for the protection of animals and were approved by the Vaud Cantonal Veterinary Office. Seven-day-old male rats (Sprague Dawley, from Janvier, France) were subjected to HI according to the Rice-Vannucci model as previously described [76]. Briefly, first, the right common carotid artery was double-ligated (Silkam, 5/0, B/BRAUN Aesculap, Kronberg im Taunus, Germany) and cut under isoflurane (3%) anesthesia. Following 2 h of recovery with the dam, the rat pups were subjected to 2 h of systemic hypoxia at 8% of oxygen in a humidified chamber maintained at 35.5 °C. Control animals used were sham-operated rat pups treated with same anesthetic and surgical procedures but without ligature and section of the common carotid artery. Nerii (0.22 mg/kg diluted in 0.5% ethanol/PBS) (Sigma, S961825) or vehicle (0.5% ethanol/PBS) was injected intraperitoneally just after carotid occlusion. For PLA experiments, rat pups were deeply anesthetized with an i.p. injection of 150 mg/kg sodium-pentobarbital, perfused transcardially with 4% paraformaldehyde in 0.1 mol/L PBS (pH 7.4) at 6 h or 24 h after HI and then cryoprotected in 30% sucrose solution. PLA experiments were performed on 18 µm thick cryostat sections. Independent experiments were conducted using rats from different litters and on different days. Animals from the same litter were divided into two groups randomly. All animals that survived hypoxia were included in the experiments, without any exclusion criteria. The number of animals per group is specified in each corresponding figure legend.

### Human newborn brain specimens

The Cantonal Ethics Commission for Research on Human Beings (CER-VD) approved experiments on human autoptic tissues. Brain tissue sections (3 µm thick paraffin sections) of autopsied human newborns (Institute of Pathology, University of Lausanne) of severe hypoxic-ischemic encephalopathy (HIE; *n* = 5) or control cases (n = 5; newborns with conditions incompatible with life whose autopsies did not indicate brain injury) were retrospectively selected from the death reports of the neonatology unit between 2001 and 2009. Autopsies were performed for medical and legal reasons, informed consent for tissue collection was obtained from the parents, and the local ethical committee approved the use of the anonymized specimens. As previously described [15, 25], perinatal data from the two groups (birth weight, gestational age, Apgar score, time of death or resuscitation score) did not show statistical differences, excepted that HIE cases showed a significantly higher metabolic acidosis (lower umbilical artery pH) and postnatal seizures compared to the control group. Control brains revealed very rare dying cells (pyknotic nuclei and shrunken cells, CASPASE3-and TUNEL-positive cells) in histological and immunohistological analyses [15, 25]. For PLA experiments, the paraffin-embedded sections were first deparaffinized and then processed for antigen retrieval as previously described [15].

### Tat-BECN1 peptide treatment

At DIV11–13 primary cortical neurons were treated with different concentrations of Tat-BECN1 (Tat-BECN1: YGRKKRRQRRRGGTNVFNATFEIWHDGEFGT, active) (Selleckchem, #S8595, Houston, TX, USA) or control peptide (Tat-sc: YGRKKRRQRRRGGVGNDFFINHETTGFATEW, inactive) (Sigma, #5310380001), a scrambled version of the C-terminal 18 amino acids from Tat–BECN1 as previously described [13]. Peptides were prepared and stored at 10 µM in acidified H_2_O (0.15% 6 N HCl). For treatment, peptides were diluted in H_2_O before to be directly added in the complete culture medium.

### Kainate-hypoxia treatment

DIV11–13 primary cortical neuronal cultures were subjected to KaHx stimulation as previously described [23]. Briefly, culture medium was removed and slowly replaced by previously deoxygenated (for 1 h) bicarbonate-buffered saline (BBS) solution (116 mM NaCl, 5.4 mM KCl, 0.8 mM MgSO_4_, 1 mM NaH_2_PO_4_, 26.2 mM NaHCO_3_, 0.01 mM glycine, 1.8 mM CaCl_2_, 4.5 mg/mL glucose [reagents from Fluka and Sigma-Aldrich]) in a sealed modular incubator chamber (Billups-Rothenberg, MIC-101, San Diego, CA, USA). Kainate (30 μM) (Enzo Life Sciences, BML-EA123-0005, Farmingdale, New York, USA) was then added and neurons were exposed to combined KaHx (6% oxygen) stimulation during the 30 min in the hypoxic chamber at 37 °C. Then, the BBS was removed and replaced with the reserved neurobasal medium mixed with one-third volume of fresh medium. Thirty min in normoxic BBS was used as the control (CTw) stimulation.

### Pharmacological treatments

Primary cortical neuronal cultures were pre-treated for 1 h with necrostatin-1 (Enzo Life Sciences, #BML-AP309-0020, Farmingdale, New York, USA) or 25 μM Q-VD-OPH (quinoline-Val-Asp(ome)-Ch2-O-phenoxy; Selleckchem, # S7311) or 50 nM Digoxin (LubioScience GmbH, # S4290-100MG, Zurich, Switzerland) or Ferrostatin-1 at 10 μM (Fer-1) (Sigma, # SML0583-5). For inhibition of lysosomal degradation, a cocktail of 10 μg/ml pepstatin A (Enzo Life Sciences, ALX-260-085-M005) and 10 μg/ml E64d (Enzo Life Sciences, BML-PI107-0001) was applied for 4 h prior to Tat-BECN1 or Tat-sc. Apoptosis was induced with staurosporin for 12 h at 1 μM (Enzo Life Sciences, # ALX-380-014-C250), necroptosis with shikonin for 6 h at 20 μM (Enzo Life Sciences, # BML-CT115-0010) and Ferroptosis with RSL3 for 24 h at 20 μM (LubioScience, #HY-100218A).

### mRFP-GFP-LC3 plasmid transfection and quantification

Primary cortical neurons plated on coverslips were transfected using Lipofectamine 2000 (Invitrogen/Thermo Fisher Scientific, 11668-019) 24 h before experiments as described previously [21]. Plasmid expressing rat LC3 fused to mRFP and EGFP, ptfLC3 (Addgene, #21074, Watertown, MA, USA), was mixed with Lipofectamine 2000 in 1:2 ratio in Neurobasal medium supplemented with only glutamine (without Penstrep and B27). After 20 min at room temperature (RT), the transfection mix was added on neurons with 2/3 of their medium replaced by Neurobasal medium supplemented with only glutamine (transfection in 0.8% B27). After 5-6 h of incubation at 37 °C, the transfection medium was removed and replaced by a mix of 1:1 old/fresh complete Neurobasal medium.

This plasmid was used to monitor the autophagy flux since it allows to discriminate between acidic (RFP^+^-GFP^-^) and nonacidic (RFP^+^-GFP^+^) LC3-positive structures (GFP is acid-sensitive whereas mRFP is not). Confocal images were acquired using a Zeiss LSM 780 Meta confocal laser-scanning microscope. Images were processed with the Zen 2.3 (blue edition) software (Carl Zeiss Microscopy GmbH, 2011, Jena, Germany) and Adobe Photoshop CC, and then analyzed using ImageJ software. The number of yellow dots (RFP^+-^GFP^+^, early autophagosomes) and of red dots only (RFP^+-^GFP^-^, autolysosomes) were counted and normalized according to the total neuron area (number of positive dots per neuron per μm^2^). The Total number of LC3-positive dots was obtained by summing the number of RFP^+-^GFP^+^ and RFP^+-^GFP^-^ dots.

### Lentiviral vectors and virus production

For knockdown experiments, shRNAs specific for rat genes from TRC (the RNAi consortium) library in pLKO lentiviral vectors (Open Biosystems/Thermo Fisher Scientific) were used as follows: a combination of TRCN0000092163 and TRCN0000092166 for *Atg7* (GenBank^TM^ NM_001012097), TRCN0000033552 for *Becn1* (GenBank^TM^ NM_053739.2), TRCN0000099431 for *Atg5* (GenBank^TM^ NM_001014250.1), TRCN0000101882 and TRCN0000101884 for ATP1a1 (GenBank^TM^ NM_012504.1), TRCN0000101780 and TRCN0000101782 for ATP1a3 (GenBank^TM^ NM_012506.1). MLKL knockdown was performed using a combination of two shRNA constructs with the following sequences: GACCAAACTGAAGACAAATAA and TCCCAACATCCTGCGTATATT (Vector Builder, Chicago, Illinois, USA) as well as for RIPK3: TCTCCAAGGTAAAGCATTATC and TCGTGAACTCGAAGAAGATAT (Vector Builder). ACSL4 knockdown was performed with a shRNA construct with the following sequence: TTACATGAAAGGGTCTTATTT (Vector Builder). BCL2 and GPX4 overexpression were achieved by self-inactivating lentiviral transfer vectors under the control of the mouse Pgk1 (phosphoglycerate kinase 1) promoter (SIN-W-PGK) transducing cDNAs sequences encoding full-length rat BCL2 or rat GPX4 (Vector Builder, NM_001039849.3). As control vectors, a pLKO vector containing scrambled shRNA (Addgene, #1864) or a SIN-W-PGK empty vectors were used. Self-inactivating lentiviral vectors were produced in 293 T cells as described previously [30]. Viral particles were resuspended in 0.1 M PBS/0.5% BSA and stored at −80 °C. Primary cortical neuronal cultures were infected between DIV5-7 and experiments were performed 5 (for downregulation) and 7 (for overexpression) days after infection.

### Quantification of cell death

#### Propidium iodide staining

After one wash in PBS-MgCl_2_ (1 mM), coverslips were incubated in PI (Sigma, #P4170) (10 μg/mL in PBS-MgCl_2_) on ice for 5 min. After two PBS-MgCl_2_ washes, neurons were fixed in 4% paraformaldehyde (PFA) in 0.1 M PBS (pH 7.4) for 15 min. Hoechst (Invitrogen/Thermo Fisher Scientific, #H3570) stain was performed (1 mg/mL in 0.1 M PBS for 5 min after one wash in PBS. After 2 more washes, coverslips were mounted in Fluorsave (Calbiochem, #345-789-20, El Cajon, CA, USA). Images were then taken using a Zeiss AxioImager imaging microscope equipped with a high-resolution camera (Axiocam 506 Color, Zeiss) using the 20× objective (EC Plan NEOFLUAR, Zeiss). At least 6 images per coverslips were taken randomly. Images were then processed with Adobe Photoshop CC and the quantification of the number of PI- and Hoechst-positive neurons was done using the Image J Fiji software. The number of PI-positive nuclei relatively to Hoechst-positive nuclei were calculated and values were expressed as a percentage of the Tat-BECN1 or KaHx values as specified in the figure legends.

#### Lactate dehydrogenase (LDH) cytotoxicity assay

Cell death was also assessed by measuring the lactate dehydrogenase (LDH) release in the medium using CytoTox 96 Non-Radioactive Cytotoxicity Assay kit (Promega, WI, USA, #G1780) as previously described [75]. Highly similar data were obtained with both methods of measuring cell death. Therefore, LDH data are not shown.

### Immunoblotting

Primary neuronal cultures were collected in lysis buffer (20 mM HEPES, pH 7.4, 10 mM NaCl, 3 mM MgCl_2_, 2.5 mM EGTA, 0.1 mM dithiothreitol, 50 mM NaF, 1 mM Na_3_Vo_4_, 1% Triton X-100 and a protease inhibitor cocktail (Sigma, #11873580001)). Cells were sonicated and protein concentration was determined by Bradford assay (Bio-Rad, #5000006, Hercules, CA, USA). Proteins (20–25 μg) were separated on SDS-PAGE (10-15% polyacrylamide (National Diagnostics, EC-890, Atlamta, GA, USA)) gels and transferred on nitrocellulose membrane (Sigma, GE10600003). Membranes were then blocked with blocking buffer (casein 0.1%, PBS 0.2%, dH_2_O, NaAzide 0.02%) at RT for about 1 h and incubated in primary antibody prepared in casein buffer at 4 °C overnight. The following primary antibodies were used for protein immunodetection: mouse monoclonal anti-actin from Millipore (#MA511869, Burlington, MA, USA, 1/2000), rabbit polyclonal anti-BCL2 from Abcam (#ab59348 Cambridge, UK, 1/1000), mouse monoclonal anti-BECN1 from Santa Cruz (#sc-48341, Dallas, Texas, USA, 1/1000), rabbit polyclonal anti-caspase3 from Cell Signaling (#9661, 1/1000), rabbit polyclonal anti-LC3 from Abcam (#ab48394, 1/1000), rabbit polyclonal anti-p62/SQSTM1 from Sigma (#P0067, 1/2000), mouse monoclonal anti-ATP1a1 from Abcam (#ab2872, 1/1000), rabbit monoclonal anti-ATP1a3 from Abcam (#ab182571, 1/1000), mouse monoclonal anti-ATP1a3 from Abcam (#ab2826, 1/1000), mouse monoclonal anti-ATG5 from Santa Cruz (#sc-133158, 1/500), mouse monoclonal anti-ATG7 from Santa Cruz (#sc-33211, 1/500). The membranes were washed with 0.1 M PBS Tween 0.1% and incubated for 1 h at RT in secondary antibody. Following secondary antibodies prepared in blocking buffer were used (at 1/10,000): goat anti-mouse IgG (H + L) DyLight 680 Conjugate (Cell Signaling, Danvers, MA, USA #5470S) and mouse anti-rabbit IgG DyLight 800 (Rockland, Pottstown, Pennsylvania, USA, #18-4516-32). After final washes, protein bands were visualized with Odyssey infrared imaging system (LI-CORE, Lincoln, NE, USA). Analyses were performed with the Odyssey v1.2 software (LI-CORE). Values were normalized relatively to the actin levels and then expressed as a percentage of Tat-BECN1- or KaHx-treated value as specified in the figure legends. Two or three culture dishes were treated per condition in each independent experiment.

### Immunoprecipitation

Immunoprecipitations were performed on hippocampal extracts (from dissected hippocampi of P7 rat sham- and HI-treated pups) and primary cortical neurons stored in lysis buffer. Five hundred μg of proteins were incubated overnight at 4 °C under agitation in 300 μL immunoprecipitation lysis buffer (25 mM TRIS (Sigma), 150 mM NaCl (Fluka), 1% NP_4_O, 1 mM Na_3_VO_4_ (Sigma), 8.4 mG NaF (Merck Fluka), anti-protease (1 stick in 500 µL H_2_O)) with 1 μg of rabbit monoclonal anti-ATP1a3 from Abcam (#ab182571) for ATP1a3 IP and mouse monoclonal anti-ATP1a1 from Abcam (#ab2872, 1/1000). 0.4 μL of IgG XP® isotype control-antibody from Cell Signaling (#3900) was used as negative control. The next day, magnetic beads (Dynabeads™ Protein G for Immunoprecipitation from Life Technologies/Thermo Fisher Scientific, #10003D) were washed in lysis buffer (supplemented with protease inhibitor cocktail (Sigma, #11873580001). The magnetic beads were then added to the samples (50 μL per protein-antibody sample) and incubated for 2 h at 4 °C under rotation. Tubes are then put on a magnet (Life Technologies/Thermo Fisher Scientific) and supernatant were taken away. 3 washes with lysis buffer were then done with 2 min of gentle rotation between each cycle. After 3 cycles, the beads were transferred in a new tube, washed again and the loading buffer (diluted 2× in lysis buffer) was added and heat for 10 min at 90 °C. Tubes were finally put on the magnet and supernatant was collected and loaded on 10% polyacrylamide gels for immunoblotting. BECN1, ATP1a1 and ATP1a3 band intensities were expressed as a ratio BECN1/ATP1a1 or BECN1/ATP1a3. The number of animals per group is specified in each corresponding figure legend.

### Immunocytochemistry

Immunostainings were performed as described previously [23] on primary cortical neurons cultured on coverslips. After one wash in PBS-MgCl_2_ (1 mM), coverslips were fixed in 4% PFA in PBS for 15 min at 4 °C. After blocking and permeabilization in PBS with 15% donkey serum (Sigma, #D9663) and 0.05% Triton X-100 for 20 min at RT, primary antibodies were diluted in PBS with 1.5% donkey serum and incubated overnight at 4 °C. The following primary antibodies were used: mouse monoclonal anti-ATP1a3 from Abcam (#ab2826, 1/100), mouse monoclonal anti-ATP1a1 from Abcam (#ab2872, 1/100), rabbit monoclonal anti-MAP2 from Millipore (#AB5622, 1/300). After 3 washes in PBS, secondary antibodies were then diluted in PBS (1/200) and incubated in the dark for 2 h at RT. The following secondary antibodies were used: Alexa Fluor 488 or 594 donkey-anti-mouse or anti-mouse (Invitrogen/Thermo Fisher Scientific, #A21202, #A21203, #A21206, #A21207). After one wash in PBS, coverslips were stained with Hoechst (2 μg/mL; Invitrogen, #H3570) in PBS for 5 min and then mounted with FluorSave (Calbiochem/Sigma, #345-789-20). Confocal microscope images of immunostained coverslips were then taken with a LSM 780 Meta confocal microscope (Carl Zeiss). Images were processed with the Zen 2.3 (blue edition) software (Carl Zeiss Microscopy GmbH, 2011), and Adobe Photoshop CC.

### Proximity ligation assay

Proximity ligation assays (PLA) were performed on primary cortical neurons, on brain slices of rat sham and HI pups and on deparaffinized brain slices from autoptic human newborns. Blocking was done in PBS with 15% donkey serum (and permeabilization with 0.3% Triton X-100 for primary cortical neurons and rat brain slices) for 30–45 min. Primary antibodies were diluted in PBS with 1.5% donkey serum and 0.1% Triton X-100 overnight at 4 °C. The Duolink® PLA Fluorescence Protocol (Sigma, #DUO92101) was followed as described by the manufacturer. After PLA staining, coverslips were stained with Hoescht (2 μg/mL; Invitrogen, #H3570) and mounted with FluorSave (Calbiochem/Sigma, #345-789-20). Confocal microscope images were processed as for immunocytochemistry. PLA-positive dots were counted using the FIJI software. Results represent the number of PLA-positive dots per neuron area in μm^2^ (for coverslips), per CA3 hippocampal neuron area in μm^2^ (for rat pup brain slices and for human newborns brain slices). The number of animals per group is specified in each corresponding figure legend.

### Electron microscopy

Primary cortical neurons were fixed 2 h in 2.5% glutaraldehyde (Electron Microscopy Sciences, #16220, Hatfield, Pennsylvania, USA) dissolved in 0.1 M phosphate buffer (PBS), pH 7.4. After 3 washes in PBS, primary cortical neurons were post-fixed for 1 h in 1% osmium tetroxide (Electron Microscopy Sciences, #19150) in PB, and then stained with ethanol 70% containing 1% uranyl acetate (Sigma, #73943) for 20 min. Primary cortical neurons were dehydrated in graded alcohol series and embedded in Epon (Electron Microscopy Sciences, #13940). Ultrathin sections of 50 nm (with silver to gray interference) were cut on a Leica Ultracut (Leica Microsystems GmbH, Wetzlar, Germany) with a diamond knife (Diatome, Biel, Switzerland) and mounted on a copper slot grid 2 × 1 mm coated with a polystyrene film. Sections were post-stained with uranyl acetate 4% in H_2_O for 10 min, rinsed several times with H_2_O followed by Reynolds lead citrate (0.2%, Sigma, #15326) in H_2_O for 10 min and rinsed several times with H2O. Micrographs were taken with a transmission electron microscope Philips CM100 (Thermo Fisher Scientific) at an acceleration voltage of 80 kV with a TVIPS TemCam-F416 digital camera (TVIPS GmbH, Gauting, Germany). For EM analyses, electron micrographs were acquired with a digital zoom ranging from 4200× to 24,500×.

#### Analyses of autophagic vacuoles number and density

ImageJ software was used to manually outline neuronal area in the digital images. For each experiment, 10 individual neurons for each condition were analyzed. The number of autophagosome/lysosome structures referred as autophagic vacuoles (AV) were counted and the density was calculated by dividing the total number of AV structures per neuron by the neuron area (in µm^2^).

#### Analyses of dying neurons with focal PNS ballooning

For each experiment, the number of dying neurons with focal PNS ballooning were counted randomly in 2 or 3 different regions (with at least 10 to 20 cortical neurons/region) and expressed as a percentage calculated by dividing the number of neurons with focal PNS ballooning by the total number of neurons counted X 100.

### Quantitative real time RT-PCR

Total mRNA was extracted using the RNeasy Mini Kit (Quiagen, #74104, Hilden, Germany). For cDNA synthesis, reverse transcriptase was carried out with High-Capacity cDNA Reverse Transcription Kit (Applied Biosystems/Thermo Fisher Scientific, #4368814) from 500 ng of total RNA. Quantitative PCR amplification were performed with Power SYBR Green PCR Master mix (BioRad, #170-8880) and 200 nM primer pair sequences (ACSL4: 5′-TATGGGCTGACAGAATCATG-3′, 5′-CAACTCTTCCAGTAGTGTAG-3′; ATP1a1: 5′-AGAAGGAAAGGGACATGCAC-3′, 5′-ATGGCTCCAATCCACAGTAA-3′: ATP1a3: 5′-GCTTTGCCTTTGACTGTGAT-3′, 5’-GATGATGCCTACACCTTTGG-3′; GPX4: 5′-AACGTGGCCTCGCAATGA-3′, 5′-GGGAAGGCCAGGATTCGTAA-3′; MLKL: 5′-CCCGAGTTGTTGCAGGAGAT-3′, 5′-TCTCCAAGATTCCATCCGCAG-3′; RIPK3: 5′-CTGTCGCCTGCTAGAGGAAG-3′, 5′-TCTGCTAACTTGGCGTGGAG-3′; GAPDH: 5′-TCCCTCAAGATTGTCAGCAA-3’, 5′-AGATCCACAACGGATACATT-3′). Samples were run in triplicate. Values were normalized with the housekeeping gene GAPDH and expressed as a percentage of shRNA *sc* transduced neurons.

### Statistical analysis

Data were expressed as mean values ± standard error of the mean (SEM). GraphPad PRISM (version 7.03) software was used for statistical analysis. The normality of the distribution was first tested using Shapiro–Wilk tests. Parametric data were analyzed using an Unpaired t-test or a Welch’s ANOVA test (one-way ANOVA with unequal variances) followed by a post-hoc Tukey’s multiple comparison test. Non-parametric data were analyzed using a Mann–Whitney test or a Kruskal–Wallis test (non-parametric analog of the one-way ANOVA) followed by a post-hoc Dunn’s multiple comparison test. Each figure legend specifies the statistical test used. p < 0.05 was chosen as threshold for statistical significance (*p < 0.05, **p < 0.01, ***p < 0.001).

### Supplementary information

Supplementary information

Supplementary table 1

Original Data File

Original Data File

## Data Availability

All data analysed during this study are included in this published article and its supplementary information files. Full and uncropped Western blots are available in the supplementary materials. All data generated during this study are available from the corresponding author upon reasonable request.
