# Supplementary material for: Neuronal autosis is Na+/K+-ATPase alpha 3-dependent and involved in hypoxic-ischemic neuronal death
Source: Cell Death Dis. 2024 May 25;15(5):363. doi: 10.1038/s41419-024-06750-2 (PMC11127954; doi:10.1038/s41419-024-06750-2)
Supplement: Supplementary file 1 — Supplementary information [file 41419_2024_6750_MOESM1_ESM.docx]

**
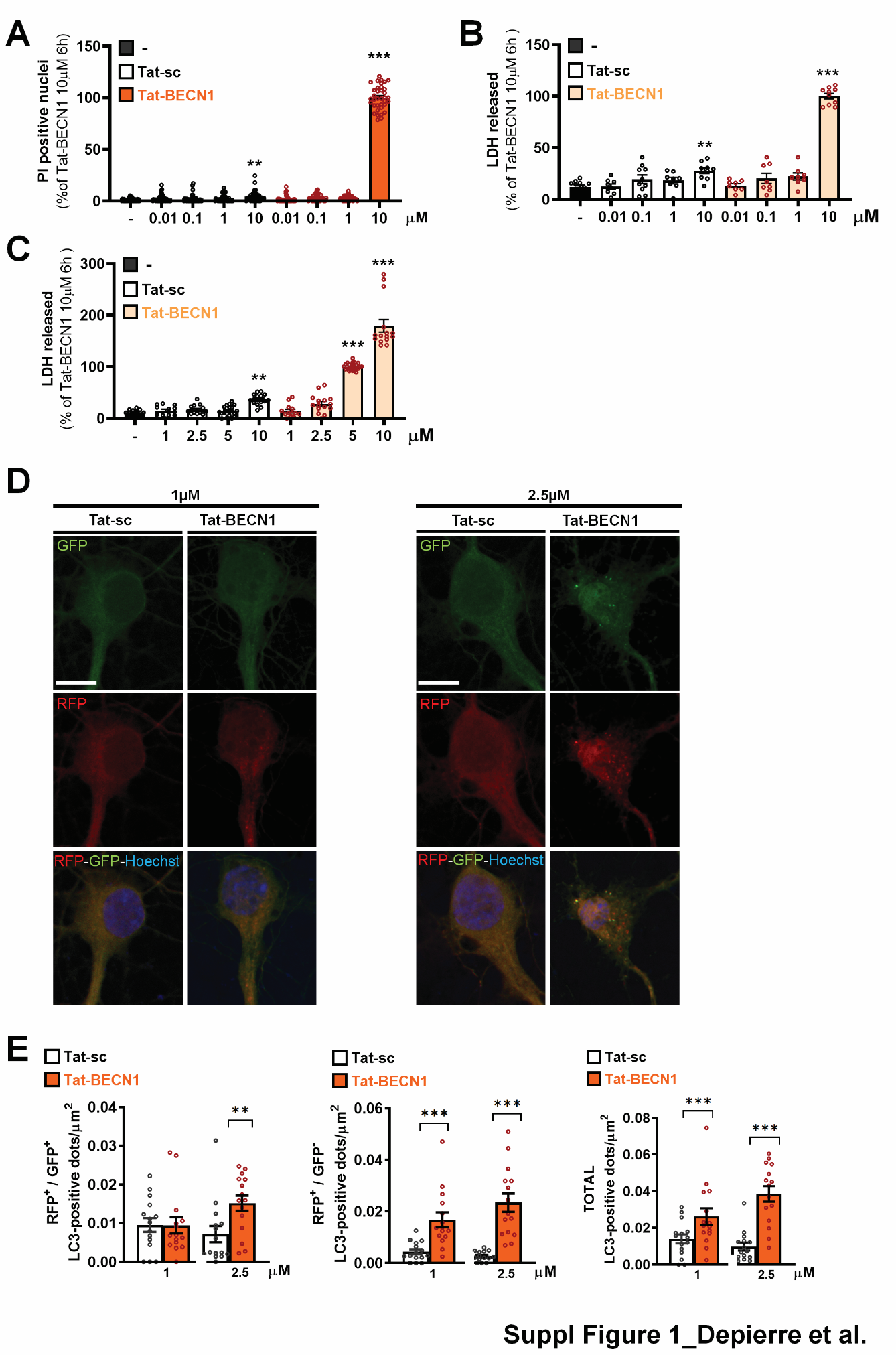
Supplementary information_Depierre et al.**

**Suppl. Figure S1. Determination of the toxic concentrations of Tat-BECN1 in primary cortical neurons and effect of non-toxic concentration on autophagy flux.**

(**A-B**) Tat-BECN1 exhibits neurotoxicity at 10 μM after 6 h when the concentration is increased logarithmically as shown by (**A**) propidium iodide (PI) staining (n=36 per concentration and 47 for untreated neurons (-), 2 independent experiments, Dunn's multiple comparisons test, compared to non-treated neurons) and (**B**) lactate dehydrogenase (LDH) assay (n≥8, 4 independent experiments, Dunnett's multiple comparisons test, compared to non-treated neurons). (**C**) Treatment with Tat-BECN1 increases LDH release at both 5 and 10 µM after 6 h compared to untreated neurons, while Tat-sc had a slight toxic effect at 10 µM. n≥ 36 per condition. 4 independent experiments. (n≥12, 8 independent experiments). Dunn's multiple comparisons test, compared to non-treated neurons. (**D**) Representative confocal microscopy images of neurons transfected with RFP-GFP-LC3 then treated with non-toxic doses of Tat-BECN1 (1 and 2.5 μM) for 3 h (scale bar = 10 µm) and (**E**) corresponding quantifications. Both the number of RFP^+^/ GFP^+^-(yellow) and RFP^+^/GFP^-^ (red) -LC3-positive dots are significantly increased in the presence of Tat-BECN1 at 2.5 μM. The total number of LC3-positive dots is also significantly increased by Tat-BECN1 at 1 and 2.5 μM. Mann-Whitney test, excepted unpaired t-test for RFP^+^/GFP^-^ -LC3 at 2.5 µM, n≥15 per condition, 3 independent experiments. Values are mean ±SEM. ** p<0.01, *** p<0.001.

**
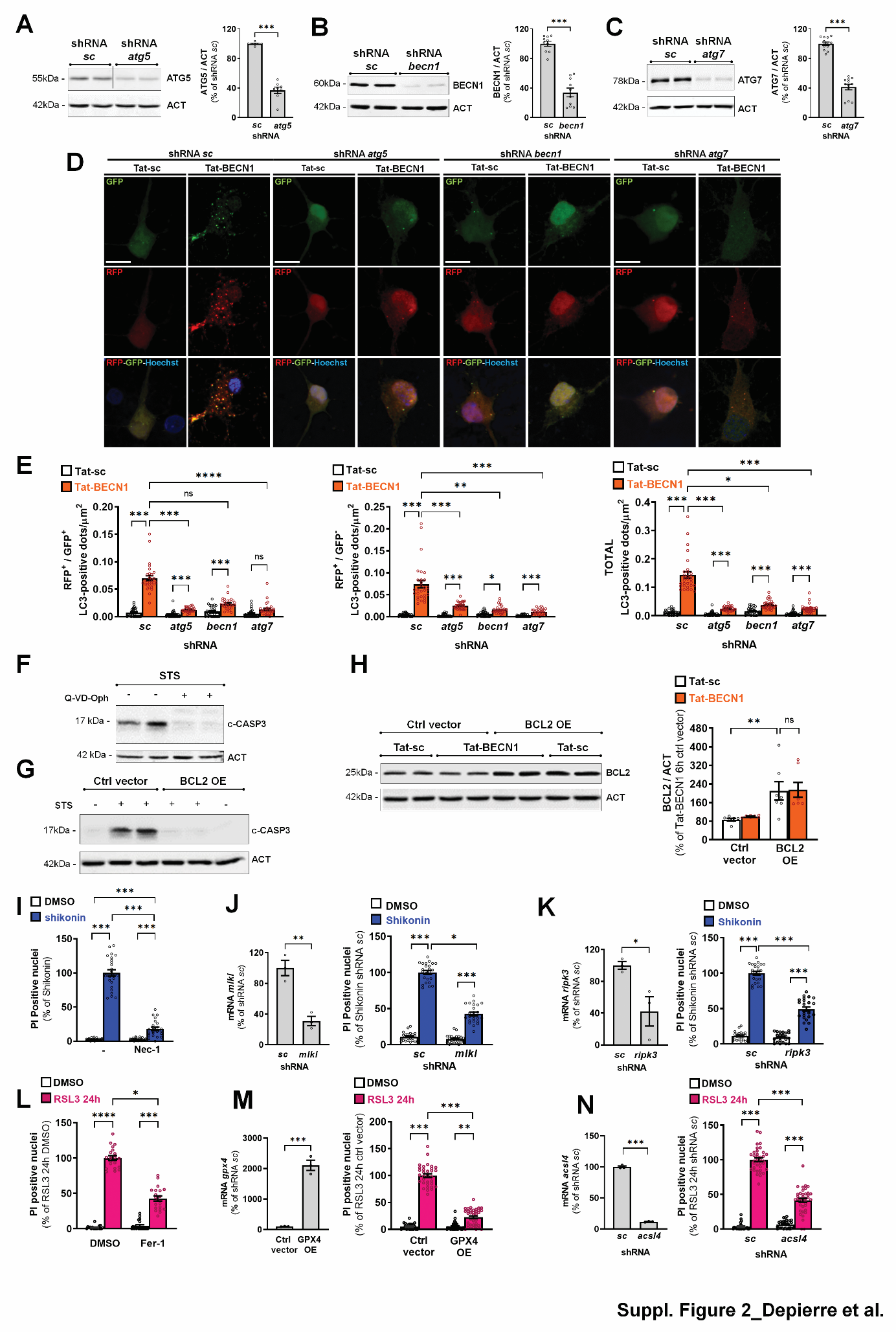
**

**Suppl. Figure S2. Tat-BECN1-induced neuronal death in primary cortical neurons is autophagy-dependent and is not involving apoptosis, necroptosis and ferroptosis.**

(**A-C**) Representative immunoblots and the corresponding quantifications of the expression of ATG proteins downregulated by lentivirus-mediated transduction of shRNAs against (**A**) *atg5* (Mann-Whitney test, n≥8 per condition), (**B**) *becn1* ( unpaired t-test, n≥8 per condition) and (**C**) *atg7* (Mann-Whitney test, n≥12 per condition). (4 independent experiments). (**D**) Representative confocal microscopy images of RFP-GFP-LC3-transfected primary cortical neurons in Tat-sc and Tat-BECN1 (5μM) conditions for each shRNAs. Scale bar = 10μm. (**E**) Downregulation of ATGs with shRNA *atg5, becn1* and *atg7* strongly reduces the increased formation of both RFP^+^/GFP^+^- (yellow, early autophagosomes) and RFP^+^/GFP^-^ (red, late autophagosome) -LC3-positive dots and of the total number of positive LC3-positive dots compared to control infected neurones (shRNA *sc*). Dunn’s multiple comparisons test, n≥30 per condition, 3 independent experiments. (**F-G**) Representative Western blots showing that (**F**) Q-VD-Oph (25µM) and (**G**) BCL2 overexpression (BCL2 OE) are efficient to completely prevent caspase-3 activation (c-CASP3) induced by 6 h of treatment with the pro-apoptotic agent staurosporine (STS, 1µM). (**H**) Representative Western blots and the corresponding quantification showing BCL2 OE efficiency and that BCL2 expression is not affected by Tat-BECN1 treatment. Dunn’s multiple comparisons test, n≥7 per condition, 3 independent experiments. (**I**) Necrostatin-1 (Nec-1, 30µM) strongly reduces the percentage of propidium iodide (PI) positive nuclei after 6 h of shikonin treatment. Tukey’s multiple comparison test, n≥24 per condition, 3 independent experiments. (**J-K**) Downregulation of (**J, left panel**) *ripk3* (n≥3 per condition) and (**K, left panel**) *mlkl* (n≥3 per condition) mRNAs after shRNA transduction significantly decrease the percentage of PI (**J and K, right panels**) positive nuclei at 6 h after shikonin treatment compared to shRNA scramble (sc) in primary cortical neurons. Dunn’s multiple comparison test for shRNA *mlkl* and Tukey’s multiple comparison test for shRNA *ripk3*, n≥24 per condition, 3 independent experiments. (**L**) Ferrostatin-1 (Fer-1, 10µM) strongly reduces the percentage of PI positive nuclei after 24 h of RSL3 (20 µM) treatment. Dunn’s multiple comparison test, n≥18 per condition, 3 independent experiments. (**M-N**) Overexpression of GPX4 (**M, left panel**) (n=3) and downregulation of (**N, left panel**) *acsl4* (n=3) mRNAs significantly decrease the percentage of PI positive nuclei at 24h h after RSL3 treatment (20 µM) in primary cortical neurons. Dunn’s multiple comparison test, n≥26 per condition, 3 independent experiments. Values are mean ±SEM. * p<0.05,** p<0.01, *** p<0.001, ns: not significant.

**
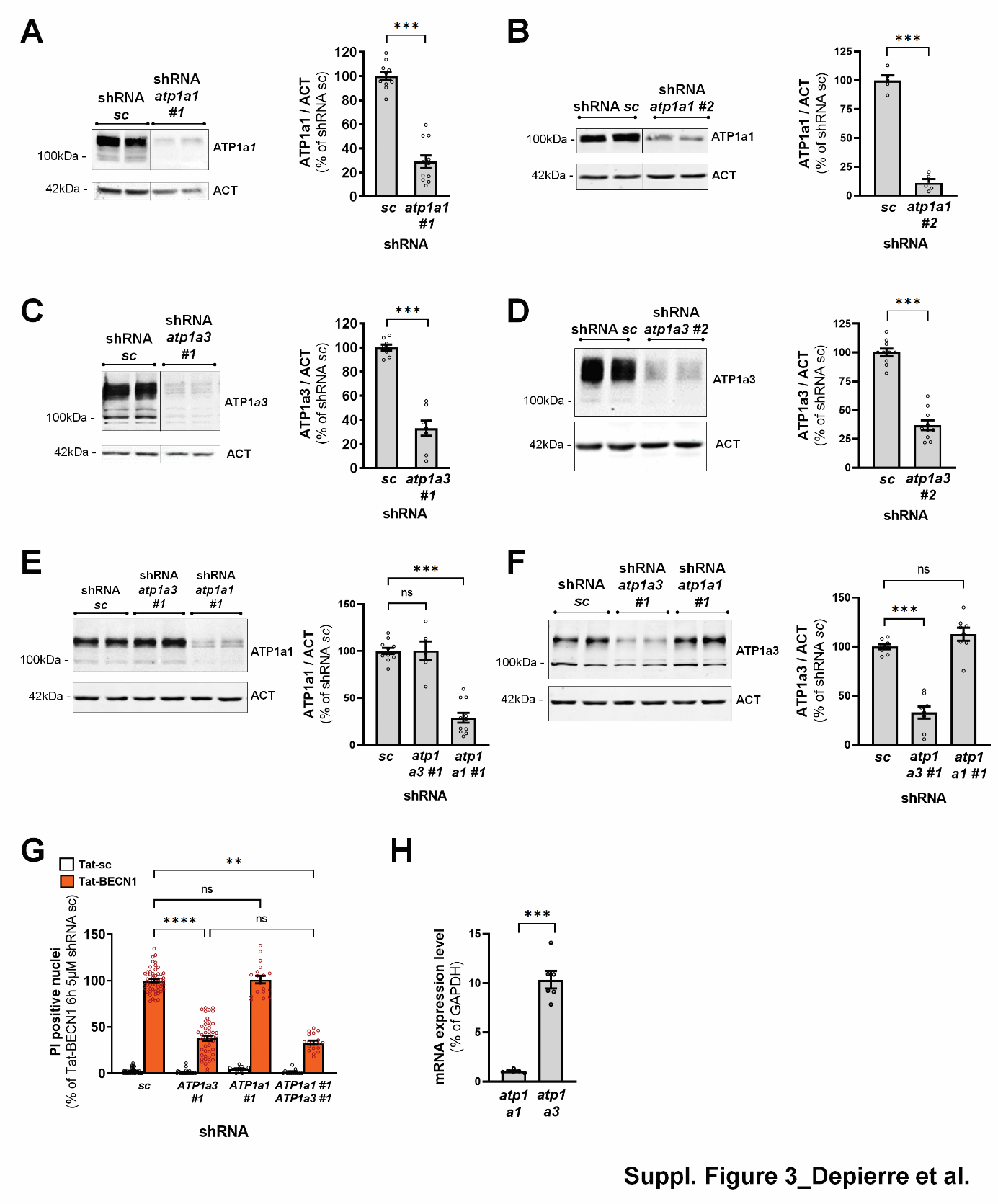
**

**Suppl. Figure S3. Tat-BECN1-induced neuronal death is ATP1a3 dependent in primary cortical neurons**

(**A-D**) Representative immunoblots and corresponding quantification showing downregulation of (**A-B**) ATP1a1 by lentiviral transduction of (**A**) shRNA *atp1a1 #1* (n≥11 per condition, 5 independent experiments) or (**B**) shRNA *atp1a1 #2* (n≥5 per condition, 3 independent experiments) and of (**C-D**) ATP1a3 with lentiviral vectors transducing (**C**) shRNA *atp1a3#1* (n≥8 per condition, 4 independent experiments) or (**D**) shRNA *atp1a3 #2* (n≥10 per condition, 7 independent experiments) in primary cortical neurons. Unpaired t-tests. (**E**) Downregulation of ATP1a1 has no effect on the expression of ATP1a3 (n≥6 per condition; 5 independent experiments) and (**F**) inversely (n≥6 per condition; 4 independent experiments). Tukey’s multiple comparison tests. (**G**) Double-knockdown of ATP1a3 and ATP1a1 does not change the neuroprotective effect of ATP1a3 downregulation at 6 h on PI-positive nuclei staining. Dunn’s multiple comparison test, n≥12 per condition 3 independent experiments. (**H**) qPCR data showing lower mRNA expression of *atp1a1* than atp1a3 mRNA expression in cortical neurons. Unpaired t-test, n=6 per condition, 1 experiment. Values are mean ±SEM. ***p<0.001, ns: not significant.

**
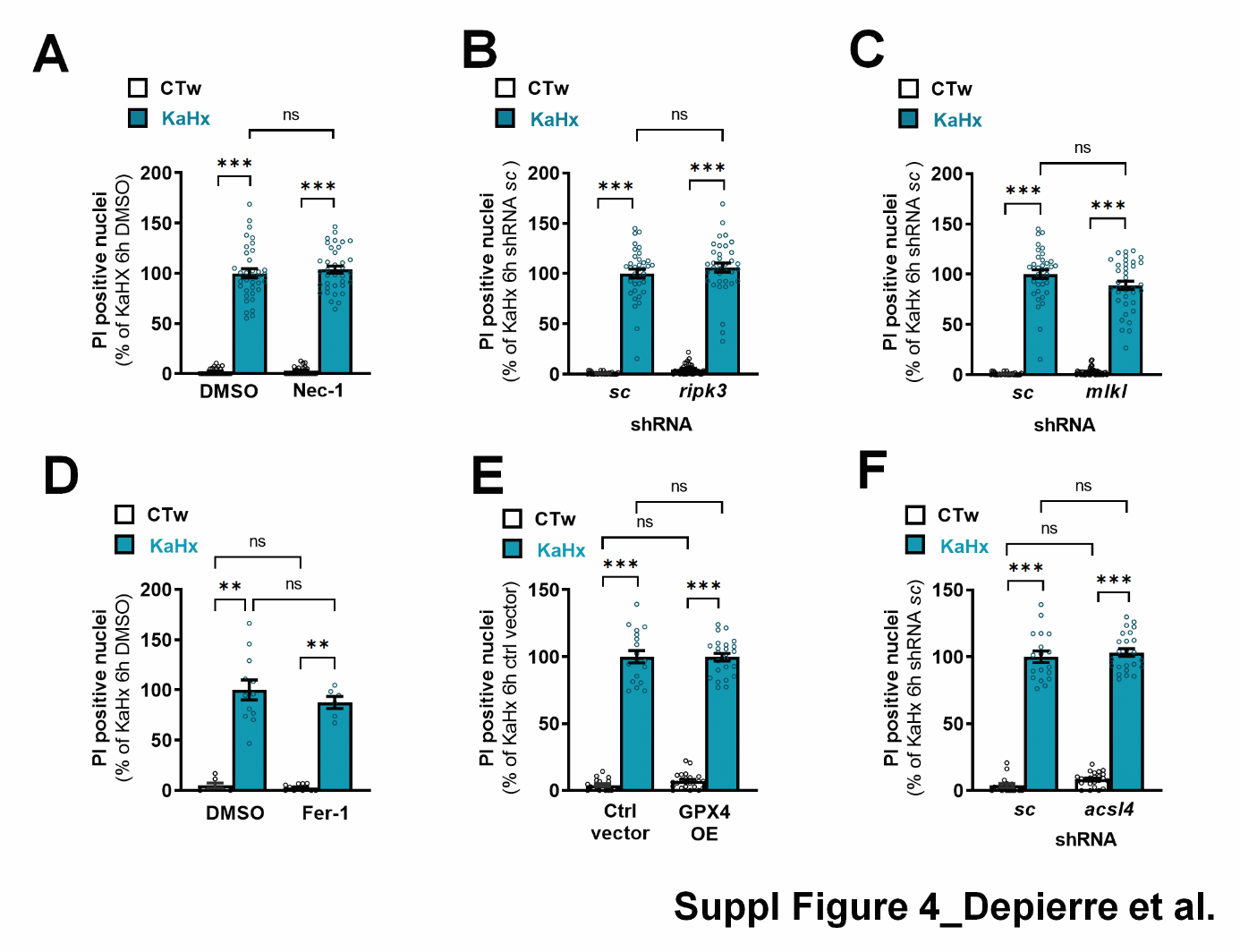
**

**Suppl Figure S4. Kainate-Hypoxia-induced neuronal death is not involving necroptosis and ferroptosis in primary cortical neurons.**

**(A-C)** Necroptosis inhibition by either **(A)** necrostatin-1 (Nec-1, 30µM) (Dunn’s multiple comparison test, n≥24 per condition, 3 independent experiments) or **(B-C)** lentiviral-mediated downregulation of **(B)** RIPK3 and **(C)** MLKL has no effect on the percentage of propidium iodide (PI)-positive nuclei after kainate-hypoxia (KaHx) compared to control (CTw). Dunn’s multiple comparison test, n=36 per condition, 2 independent experiments. **(D-F)** Ferroptosis inhibition by either **(D)** ferrostatin-1 (Fer-1, 10 µM) (Dunn’s multiple comparisons test, n≥6 per condition, 3 independent experiments) or with lentiviral-mediated **(E)** overexpression of GPX4 (GPX4 OE) (or **(F)** knockdown of ACSL4 does not reduce KaHx-induced increase of PI-positive nuclei. Dunn‘s multiple comparisons test, n≥18 per condition, 3 independent experiments. Values are mean ±SEM. **p<0.01, ***p<0.001, ns: not significant.

**
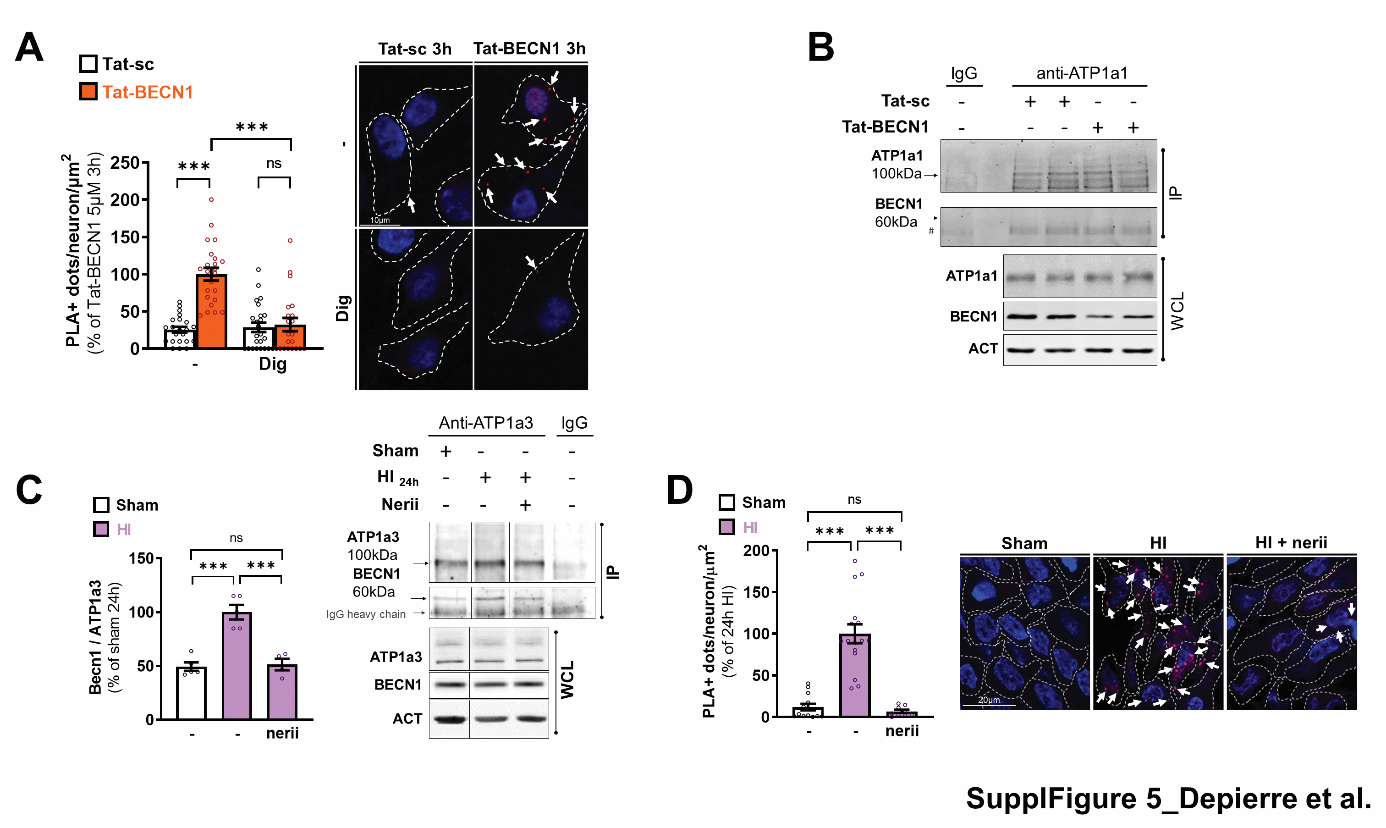
Suppl. Figure 5. ATP1a3-BECN1 interaction induced by autosis is prevented by cardiac glycosides.**

**(A)** Representative images and corresponding quantifications of proximity ligation assays (PLAs) of BECLIN1 (BECN1) and ATP1a3 in primary cortical neurons treated with Tat-BECN1 or Tat-sc for 3h with or without Digoxin (Dig, 50 nM) treatment (n≥21, Dunn’s multiple comparison test; 2 independent experiments; scale bar = 10 µm). **(B)** Representative immunoblots of a coimmunoprecipitation of BECLIN1 (BECN1) with ATP1a1 in primary cortical neurons treated with Tat-BECN1 or Tat-sc for 3h showing that BECN1 does not interact with ATP1a1 in Tat-BECN1 conditions. **(C)** Representative immunoblots and corresponding quantifications of coimmunoprecipitation of BECN1 with ATP1a3 in hippocampus samples of rat pups after 24 h of hypoxia-ischemia (HI) and in corresponding sham animals showing that neriifolin (nerii, 0,22 mg/kg) prevents BECN1-ATP1a3 interaction induced in HI conditions (n≥4 animals/condition, Tukey’s multiple comparison test, 3 independent experiments). **(D)** Representative images and corresponding quantifications of PLAs of BECN1 and ATP1a3 in CA3 hippocampus region of rat pups after 24 h of hypoxia-ischemia (HI) (and in corresponding sham animals) showing that nerii treatment prevents the HI-induced increase in PLA-positive dots (n≥9 images analysed from 4 animals/conditions, Dunn’s multiple comparison test, 3 independent experiments, scale bar = 20 µm). Values are mean ±SEM. ***p<0.001, ns: not significant.
