# Supplementary material for: Neuronal autosis is Na+/K+-ATPase alpha 3-dependent and involved in hypoxic-ischemic neuronal death
Source: Cell Death Dis. 2024 May 25;15(5):363. doi: 10.1038/s41419-024-06750-2 (PMC11127954; doi:10.1038/s41419-024-06750-2)

Full unedited gels; Depierre P. et al.

Full unedited gels for Figure 1D

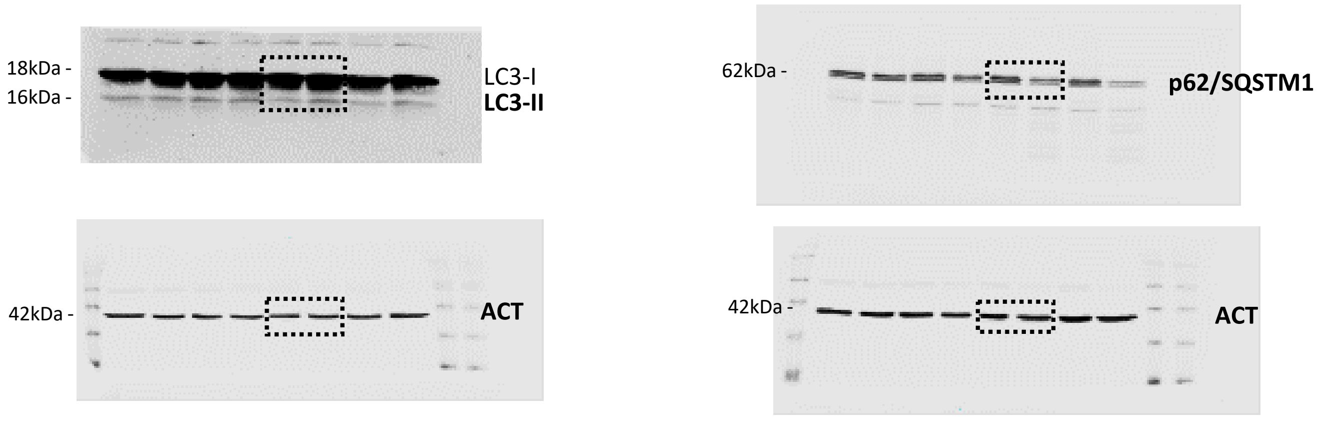

Full unedited gels for Figure 1E

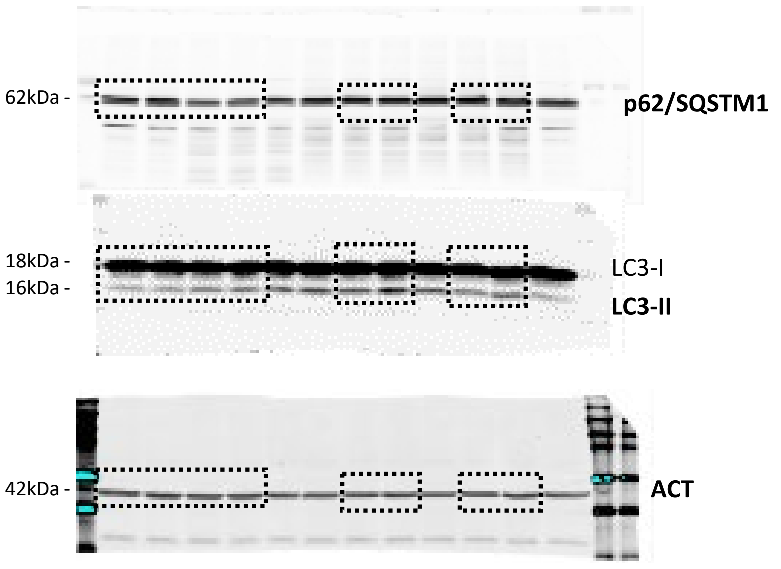

Full unedited gels for Figure 6A

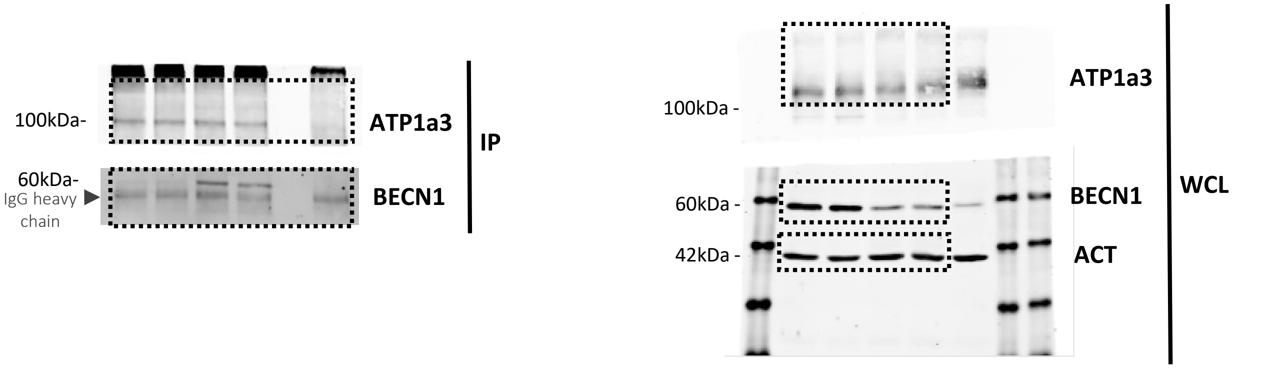

Full unedited gels for Figure 6D

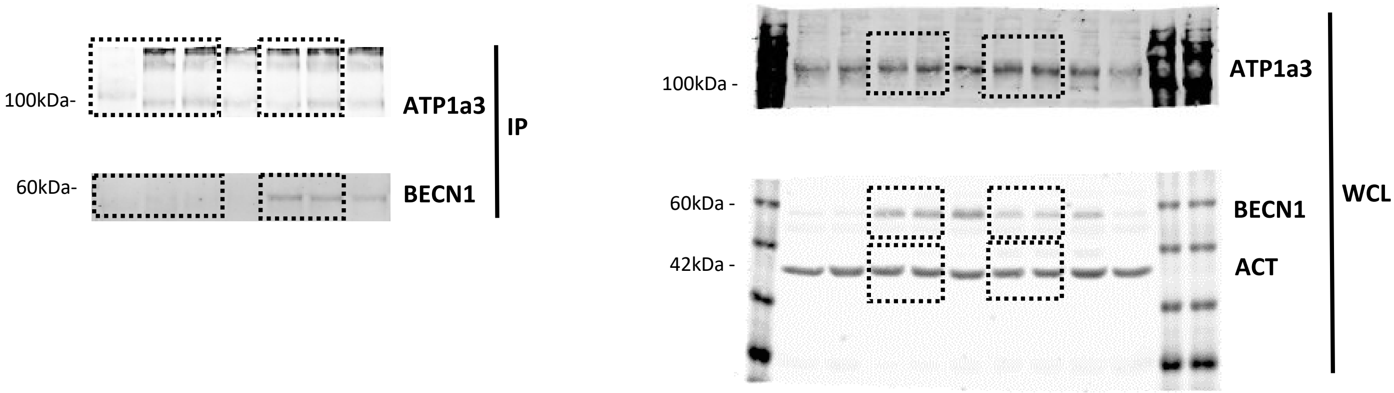

Full unedited gels for Figure 6G

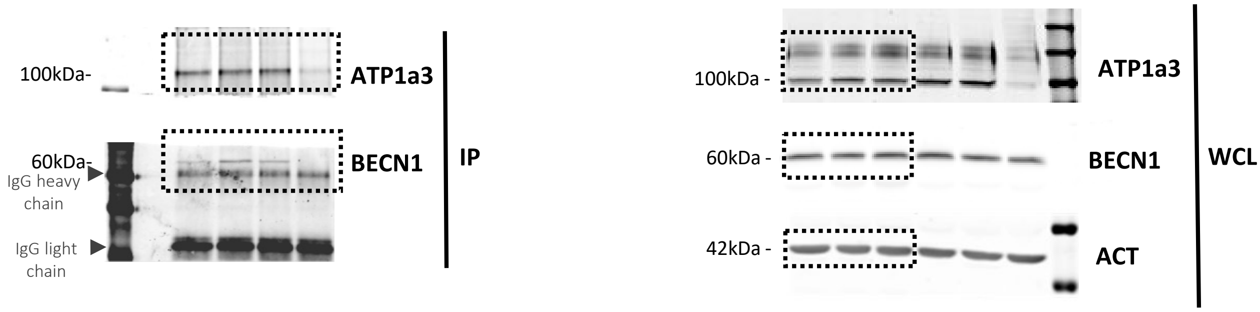

Full unedited gels, Supplementary Data; Depierre P. et al.

Full unedited gels for Figure S2A

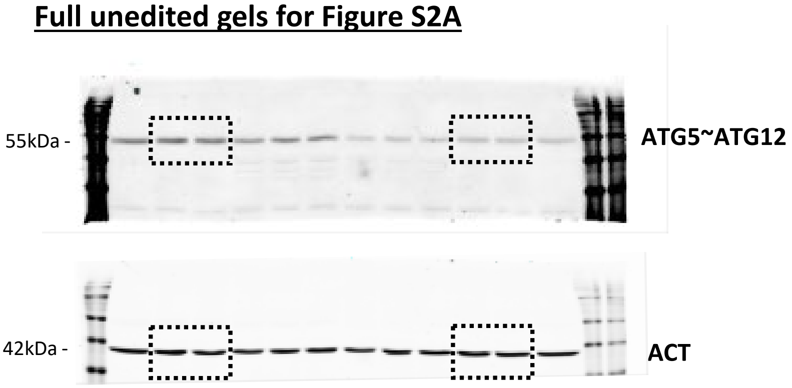

Full unedited gels for Figure S2B

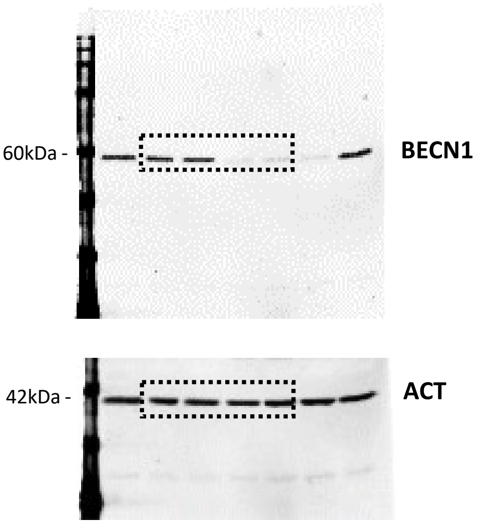

Full unedited gels for Figure S2C

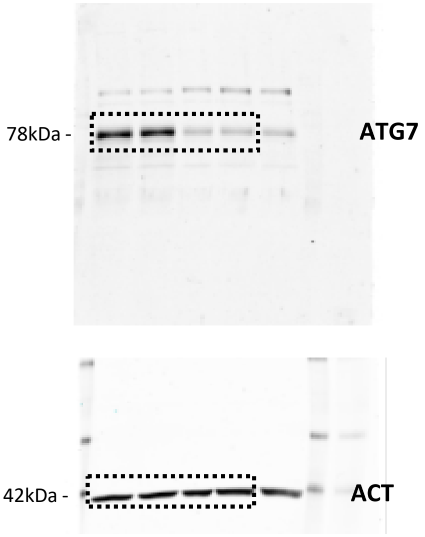

Full unedited gels for Figure S2F

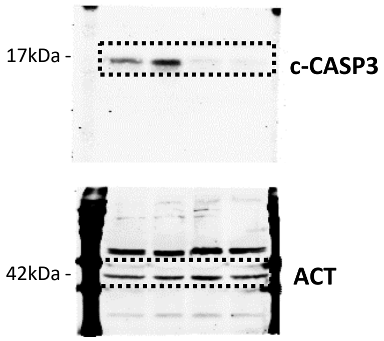

Full unedited gels for Figure S2G

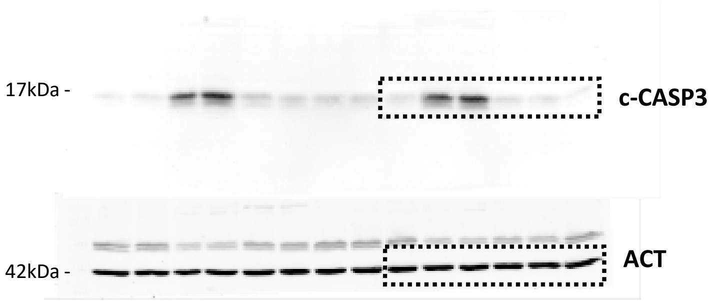

Full unedited gels for Figure S2H

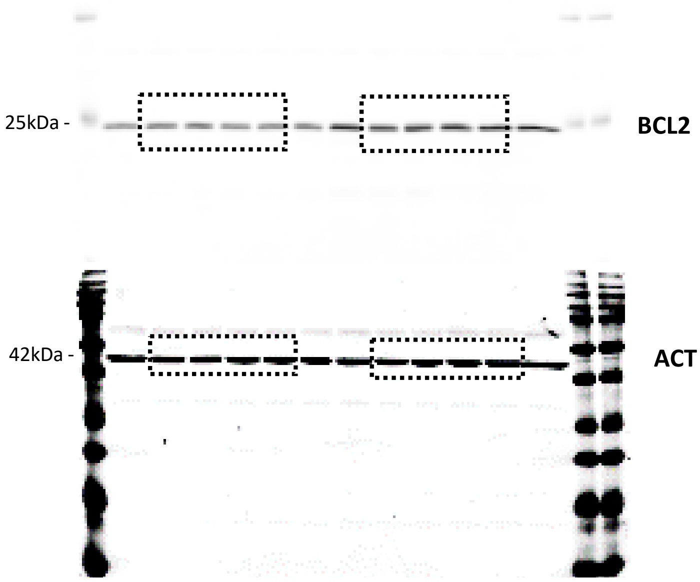

Full unedited gels for Figure S3A

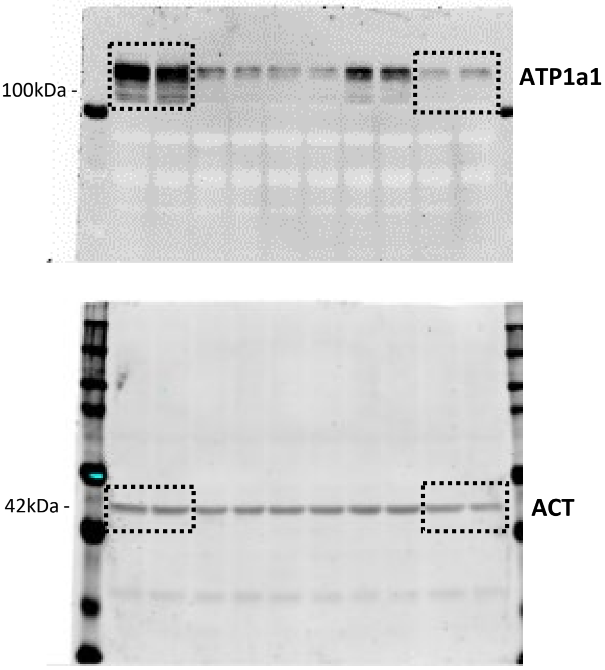

Full unedited gels for Figure S3B

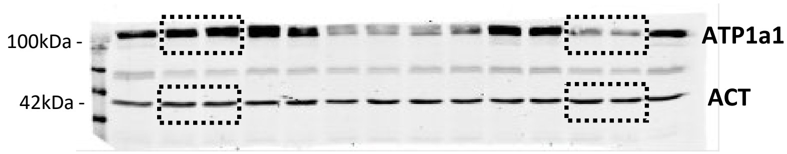

Full unedited gels for Figure S3C

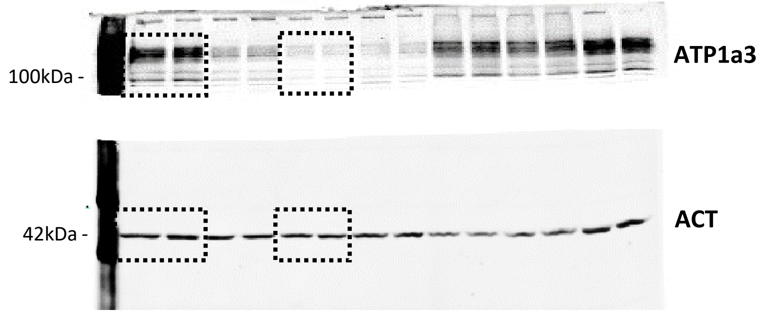

Full unedited gels for Figure S3D

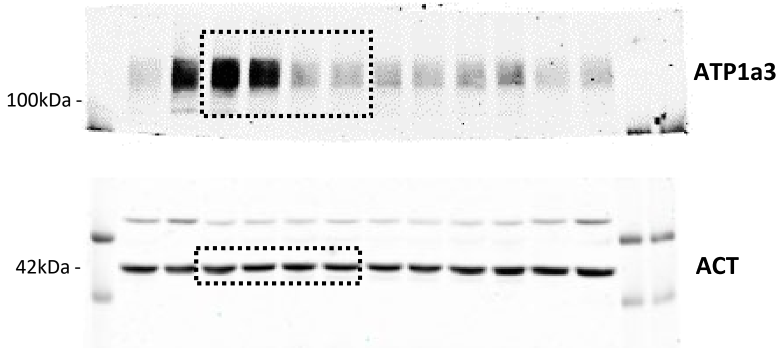

Full unedited gels for Figure S3E

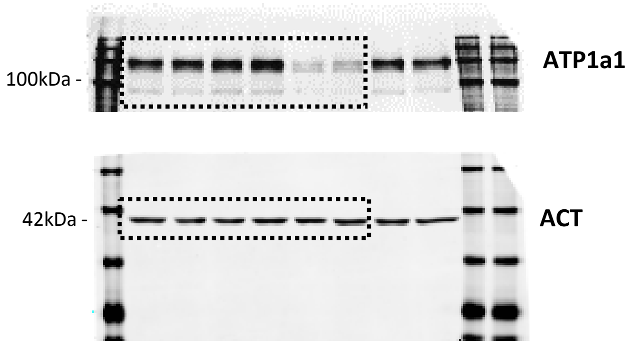

Full unedited gels for Figure S3F

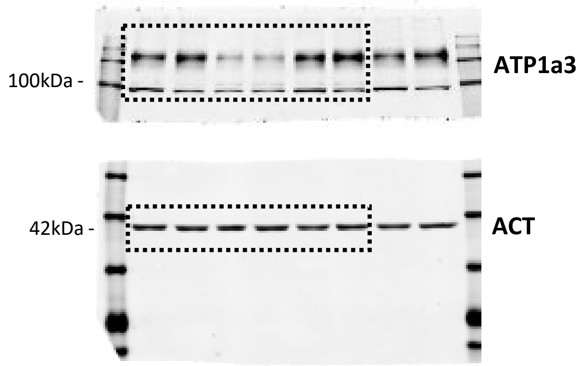

Full unedited gels for Figure S5B

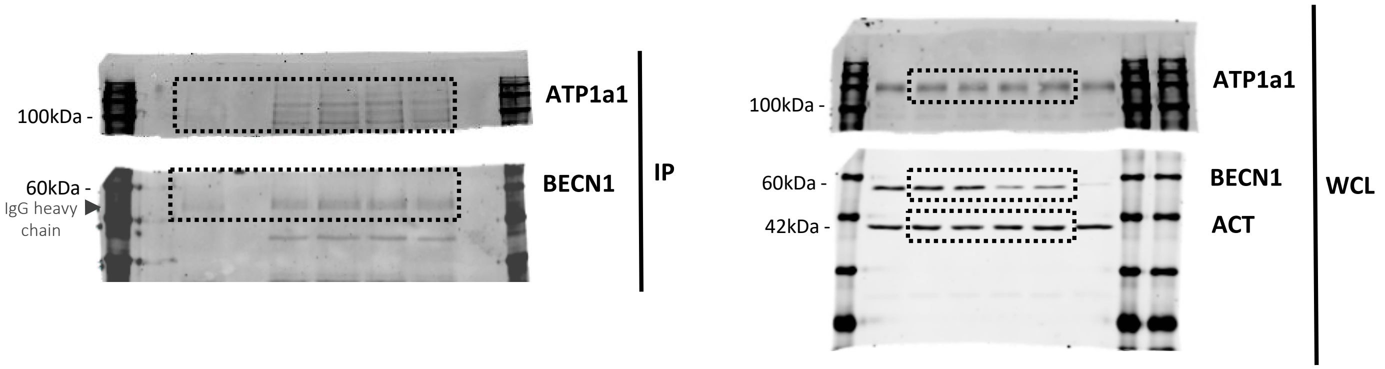

Full unedited gels for Figure S5C

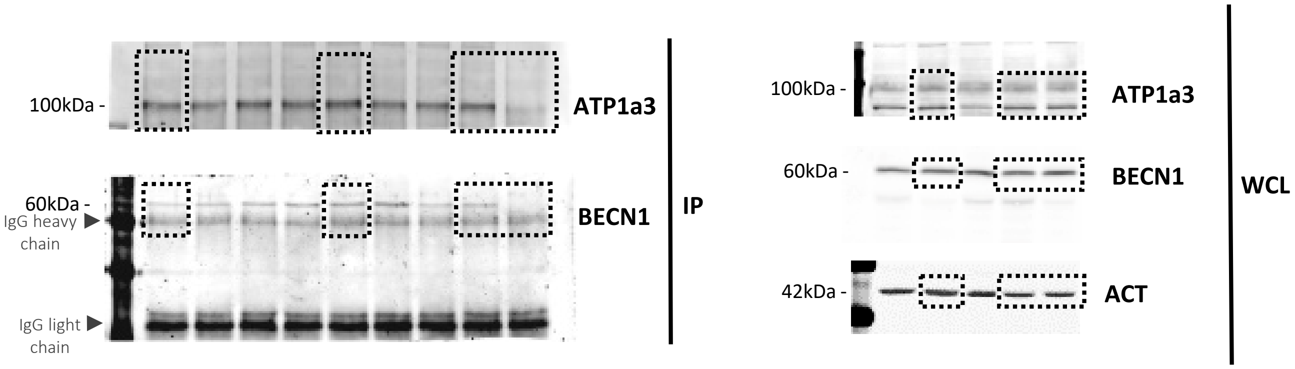

Supplement: Supplementary file 3 — Original Data File [file 41419_2024_6750_MOESM3_ESM.pdf]
